# Single-cell RNA sequencing reveals the suppressive effect of PPP1R15A inhibitor Sephin1 in antitumor immunity

**DOI:** 10.1016/j.isci.2023.105954

**Published:** 2023-01-13

**Authors:** Rongjing Wang, Yuchao Zhang, Shiwei Guo, Siyu Pei, Wei Guo, Zhenchuan Wu, Hailong Wang, Minghui Wang, Yizhe Li, Yufei Zhu, Ling-Hua Meng, Jingyu Lang, Gang Jin, Yichuan Xiao, Landian Hu, Xiangyin Kong

**Affiliations:** 1 Shanghai Institute of Nutrition and Health, Chinese Academy of Sciences, CAS Key Laboratory of Tissue Microenvironment and Tumor, Shanghai, China; 2 Changhai Hospital, Department of Hepatobiliary Pancreatic Surgery, Shanghai, China; 3 Department of Thoracic Surgical Oncology, Shanghai Lung Cancer Center, Shanghai Chest Hospital, Shanghai Jiao Tong University School of Medicine (SJTUSM), Shanghai 200030, China; 4 University of Chinese Academy of Sciences, Shanghai, China; 5 ShanghaiTech University, School of Life Science and Technology, Shanghai, China; 6 Division of Anti-tumor Pharmacology, Shanghai Institute of Materia Medica, Chinese Academy of Sciences, Shanghai, China; 7 Anda Biology Medicine Development (Shenzhen) Co, Ltd, Shanghai, China

**Keywords:** Biological sciences, Molecular biology, Immunology, Transcriptomics

## Abstract

Protein phosphatase 1 regulatory subunit 15A (PPP1R15A) is an important factor in the integrated stress response (ISR) in mammals and may play a crucial role in tumorigenesis. In our studies, we found an inhibitor of PPP1R15A, Sephin1, plays a protumorigenic role in mouse tumor models. By analyzing the single-cell transcriptome data of the mouse tumor models, we found that in C57BL/6 mice, Sephin1 treatment could lead to higher levels of ISR activity and lower levels of antitumor immune activities. Specifically, Sephin1 treatment caused reductions in antitumor immune cell types and lower expression levels of cytotoxicity-related genes. In addition, T cell receptor (TCR) repertoire analysis demonstrated that the clonal expansion of tumor-specific T cells was inhibited by Sephin1. A special TCR + macrophage subtype in tumor was identified to be significantly depleted upon Sephin1 treatment, implying its key antitumor role. These results suggest that PPP1R15A has the potential to be an effective target for tumor therapy.

## Highlights

• Inhibition of PPP1R15A can lead to antitumor immunity suppression
• Complete the single-cell transcriptome and TCR profiling of B16F1 tumor immune cells
• TCR + macrophages can have important functions in antitumor immunity

## Graphical abstract

**Figure:**
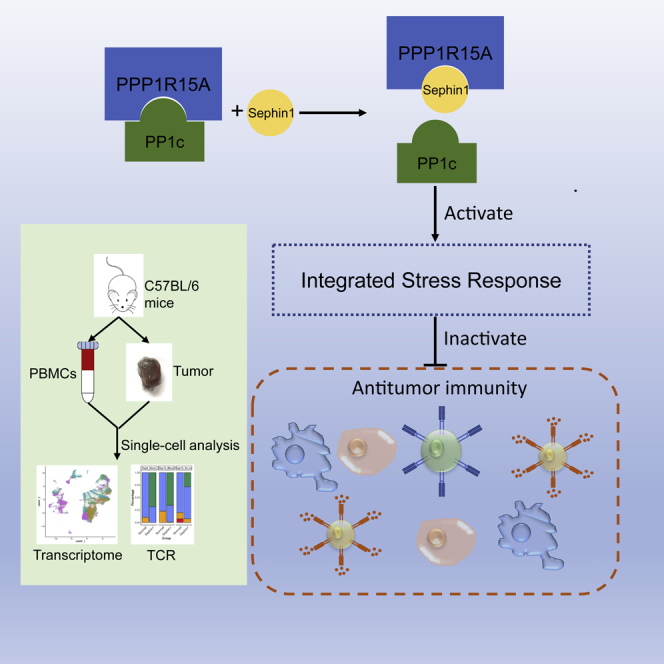

## Introduction

Immunotherapy has become the most promising treatment for many cancers because of its unique advantages related to improved tumor specificity and reduced side effects. The development of immunotherapy has depended on research on the tumor immune microenvironment (TIME),^1^ including research on cytokines, interferons, and interleukins for nonspecific immunostimulatory therapy^2^^,^^3^; studies developing approaches for the transfer or stimulation of T cells and natural killer (NK) cells for specific immunotherapy^4^^,^^5^; research on regulatory T cells for checkpoint-based immunotherapy^6^; and studies on dendritic cell (DC)-based immunotherapy.^7^ Some kinds of lymphocytes are crucial in antitumor immune activities, including cytotoxic T cells, which recognize tumor cells and perform precise cytotoxic functions,^8^^,^^9^ and NK cells, which kill tumor cells independent of antigen presentation.^10^^,^^11^^,^^12^ Some myeloid cells are also important in the TIME, including DCs and tumor-associated macrophages (TAMs).^13^^,^^14^ Because of the complexity of the TIME and the heterogeneity among different tumor types, there is still much to be explored regarding the regulatory mechanism and related cytokines in the tumor microenvironment. Therefore, understanding the roles of different cells in the TIME and identifying the key genes and proteins that affect their functions are very important for cancer immunotherapy.

Protein phosphatase 1 regulatory subunit 15A (PPP1R15A), which is also known as growth arrest and DNA damage-inducible protein 34 (GADD34),^15^ is a key factor in an essential biological process in mammalian cells, the integrated stress response (ISR).^16^ As an evolutionarily conserved process, the ISR can be coupled to both the unfolded-protein response (UPR) and the Heme-regulated eukaryotic translation initiation factor 2α (eIF2α) kinase (HRI) activation^17^^,^^18^ and is activated by both the endoplasmic reticulum (ER) and cytosolic lumen,^19^ which makes this process crucial for cells, tissues, and organisms to adapt to variable environments and maintain homeostasis.^20^ PPP1R15A can bind to catalytic subunit protein phosphatase 1 (PP1c) and promote the dephosphorylation of eIF2α,^21^^,^^22^ which regulates the ISR through its phosphorylation and dephosphorylation process.^20^^,^^23^ When eIF2α is phosphorylated, global protein synthesis is reduced, and the translation of activating transcription factor 4 (ATF4) is activated,^24^ which benefits cell survival and recovery. In contrast, the dephosphorylation of eIF2α allows the cell to recover normal protein synthesis processes.^25^^,^^26^ ATF4 can bind to a basic region/leucine zipper motif (bZIP) transcription factor, namely, CCAAT/enhancer binding protein (C/EBP) homologous protein (CHOP),^27^^,^^28^ to form the ATF4-CHOP complex. The ATF4-CHOP complex plays an important role in mammalian autophagy, including the induction of autophagy and activation of autophagy-related genes,^28^^,^^29^ such as activating trasncription factor 3 (ATF3), PPP1R15A, and Tribbles pseudokinase 3 (TRIB3).^30^

Because of the importance of the ISR in mammalian cells, many cell types and diseases can be affected by this process. Several studies have suggested that the ISR functions in cognitive and neurodegenerative disorders,^31^^,^^32^^,^^33^ metabolic disorders,^34^^,^^35^ and cancers.^36^^,^^37^ Previous studies have found that the influence of the ISR on cancers can be dual because the induced hypoxia can either induce tumor apoptosis or promote the growth of tumor cells that tolerate hypoxia.^38^^,^^39^ In addition, the ISR also has a significant influence on mammalian immunity. Studies have shown that the ISR can affect the innate immune response^40^^,^^41^ and the secretion of some kinds of cytokines, including interleukin-1β (IL1β) and interleukin-6 (IL6).^42^^,^^43^ These influences may be highly dependent on the phosphorylation and dephosphorylation of the eIF2 complex,^44^ especially the eIF2α subunit. On the other hand, the activation of ATF4 is also important in immune-related processes.^45^

Because of the key roles of the ISR in cancer and immunity, we decided to investigate the relationship between the ISR and antitumor immune activities. The dephosphorylation of eIF2α is performed by the combined influence of the PPP1R15A-PP1c complex and PPP1R15B-PP1c complex. However, PPP1R15B functions in a steady and constitutive way,^46^ while PPP1R15A is part of a feedback mechanism that antagonizes the relative strength of ISR activation.^25^ Therefore, the selective suppression of the function of PPP1R15A could be safer,^47^ and selective inhibitors of PPP1R15A are better regulators of the ISR process in cells.

Based on these facts, we chose a selective inhibitor of PPP1R15A, Sephin1, to conduct our experiments. Sephin1 can selectively bind to PPP1R15A, thus inhibiting the formation of the PPP1R15A-PP1c complex without affecting the function of PPP1R15B, which makes it safer for use in animals.^48^ Because of its effect to PPP1R15A and ISR, Sephin1 is now used as a potential treatment in neuron-, motor-, and proteostasis-related diseases.^48^^,^^49^^,^^50^ In this study, we used Sephin1 as an inhibitor of PPP1R15A to explore the effect of Sephin1 on the TIME, especially the impact on antitumor immune cells. We performed both *in vivo* and *in vitro* experiments and studied two different cell lines, B16F1 and 4T1. By single-cell sequencing of C57BL/6 mice implanted with B16F1 cells, we found that the influence of Sephin1 on the mouse tumor microenvironment was complicated and that an immunosuppressive effect impacted multiple immune cell types. The protumorigenic effect of Sephin1 was also verified in BALB/c mice implanted with 4T1 cells. In addition, *in vitro* experiments proved the suppressive effect of Sephin1 on mouse Cd8+ T cells. These results all indicate that Sephin1 can suppress antitumor immune activities in different tumor types, which implies that PPP1R15A has potential as a target in tumor immunotherapy.

## Results

### Sephin1 accelerates tumor progression with ISR activation and immune response suppression

**Figure 1:**
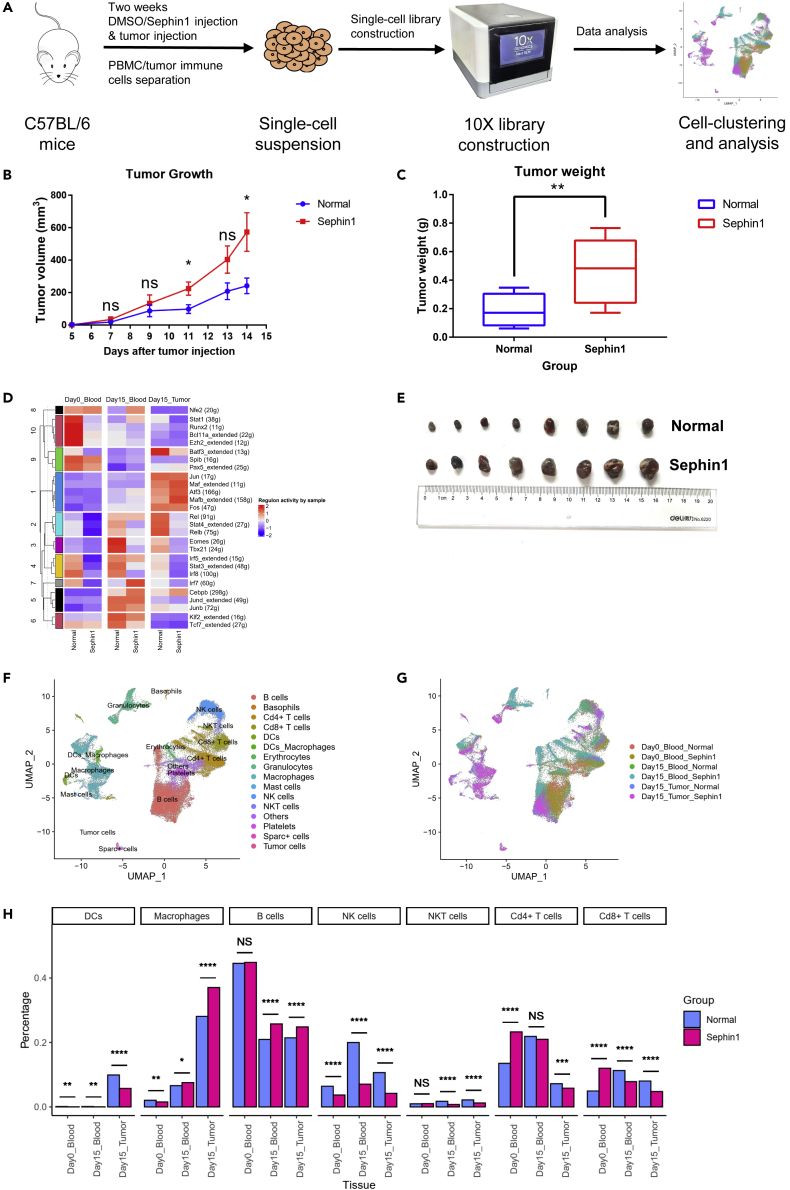
Differences in tumor development and regulon activities and cell distribution between the normal and Sephin1 groups (A) Overall experimental design. Mice were first separated into the normal group and Sephin1 group and injected with solvent or Sephin1 for two weeks. Then, B16F1 cells were injected. PBMCs were collected on days 0 and 15 after tumor injection, and immune cells were isolated from tumor tissues on day 15 and subjected to single-cell sequencing. (B) Tumor growth curves of the normal group and Sephin1 group (n = 8). The tumor volume in the Sephin1 group was significantly higher than that in the normal group. Multiple t tests were used without adjustments, and each row was analyzed individually. Bars: mean; error bars: SEM; ∗: p < 0.05. (C) Tumor weights of the two groups (n = 8) on day 15. The tumor weight of the Sephin1 group was significantly higher. Unpaired, two-tailed t test was used. Bars: mean; error bars: SEM; ∗∗: p < 0.01. (D) SCENIC analysis based on the single-cell sequencing data for different samples. The Atf3 regulon had higher activity in the Sephin1 group in all three sample types. The number of genes in each regulon is shown in brackets. (E) Tumor images from the normal (top) and Sephin1 (bottom) groups. (F) Cell type annotation of all 12 samples. Sixteen cell types were identified. (G) Cell distribution of all 6 sample types (2 samples of each type). (H) Distribution of major cell types in different sample types. Day 0_Blood—PBMCs in blood samples collected on day 0. Day 15_Blood—PBMCs in blood samples collected on day 15. Day 15_Tumor—Cd45 + immune cells in tumor tissues collected on day 15. Chi-squared test was used to evaluate the distribution of normal/Sephin1 groups. ∗: p < 0.05; ∗∗: p < 0.01; ∗∗∗: p < 0.001; ∗∗∗∗: p < 0.0001.

Three types of samples including peripheral blood mononuclear cells (PBMCs) harvested on day 0 (the day of tumor cell injection) and day 15 (15 days after tumor cell injection) and immune cells isolated from tumor tissues harvested on day 15 were collected for single-cell sequencing (Figure 1A). Tumor growth curves showed that the tumor growth rate in the Sephin1 group was significantly higher than that in the normal group (Figure 1B). Tumor tissues taken from both groups on day 15 demonstrated that the tumor volume and weight in the Sephin1 group were also significantly higher than those in the normal group (Figures 1C and 1E). Apart from these findings, we repeated the *in vivo* Sephin1 experimental procedure with female BALB/c mice implanted with the mouse triple-negative breast cancer cell line 4T1. The tumor growth rate of 4T1 cells in the Sephin1 group was also higher than that in the normal group, indicating that the suppressive effect of Sephin1 on antitumor immunity may be common among different tumor types. However, the result was not as significant as that for the B16F1 cell line in male C57BL/6 mice (Figure S10C), implying that the mechanism underlying the difference between the two cell lines needs to be further explored.

Raw single-cell sequencing data were first analyzed using CellRanger software developed by 10X Genomics and then merged, filtered, and clustered with Seurat. After quality control, 68,531 cells from 12 samples were included (Table S1). The 12 samples were analyzed by sample type, and the samples included the normal and Sephin1 samples of blood collected on day 0 and day 15 and tumor immune cell samples collected on day 15, for a total of 6 sample types. Single-cell regulatory network inference and clustering (SCENIC) analysis was performed and used to compare the samples to analyze regulon activity. A regulon is a coexpression module with significant motif enrichment of a certain upstream regulator, and higher regulon activity reveals higher *cis*-regulatory activity.^51^ Twenty-seven regulons were identified in all six sample types, and the regulons were separated into 10 clusters by K-means based on their activity profiles (Figure 1D). The Atf3 regulon, which includes genes involved in the ISR, such as Atf4 and Atf3, and related regulatory genes, had higher activity levels in the Sephin1 group blood samples collected on day 0 and day 15 and tumor samples collected on day 15. Regulons in the same cluster as Atf3, including Jun, Maf_extended, Mafb_extended, and Fos regulons, had activity profiles same as to that of the Atf3 regulon. However, regulons related to immune cell activities and differentiation, such as Stat1-, Stat3-, and Stat4-related regulons, were downregulated in the Sephin1 group. In addition, regulon specificity score (RSS) analysis also indicated that the Atf3 regulon was the most specific regulon in the day 15 tumor samples (Figure S1B).

### Sephin1 causes suppression of antitumor immunity mediated by multiple immune cell types

To further explore the modulatory effect of Sephin1 on the immune microenvironment, we analyzed the compositional changes in the broad categories of immune cells in the peripheral blood and tumors. Cells from all 12 samples were first separated into 6 sample types (Figures 1G and S1C) and then preprocessed, integrated, and clustered with Seurat, and a total of 58 clusters were identified (Figure S1A). These clusters were classified as different cell types based on classic cell markers, and all cells were separated into 16 types, including 5 lymphocyte cell types (NK cells, natural killer T (NKT) cells, Cd8+ T cells, Cd4+ T cells, and B cells) and 6 myeloid cell types (Basophils, Granulocytes, DCs_Macrophages, Macrophages, DCs, and Mast cells; Figure 1F). The marker gene expression profiles of different cell types are also shown (Figure S2A). In the Uniform Manifold Approximation and Projection (UMAP) graph grouped by sample (Figure 1G), we saw that the clustering between samples did not have a significant batch effect, which demonstrated that the sequencing data were comparable among different samples. The percentages of different cell types were calculated and analyzed (Figures 1H and S2B), and different samples showed different cell type distribution profiles. The cell types related to antitumor immune activities were more likely to be reduced in the Sephin1 group, especially in the tumor microenvironment on day 15, including Cd8+ T cells, NKT cells, NK cells, and DCs. However, the distribution of macrophages was more likely to be enriched in the Sephin1 group. Subtype analysis was further performed on these important immune cell types.

Gene set variation analysis (GSVA) was also performed to compare the different sample types (Figure S1D). In the tumor samples, genes expression level related to the melanin biosynthetic process, the cellular response to amino acid stimulus and adenosine triphosphate (ATP) hydrolysis-coupled proton transport was upregulated in the Sephin1 group, which indicated higher ISR levels.

### Sephin1 can lead to a significant reduction in antitumor lymphocytes

**Figure 2:**
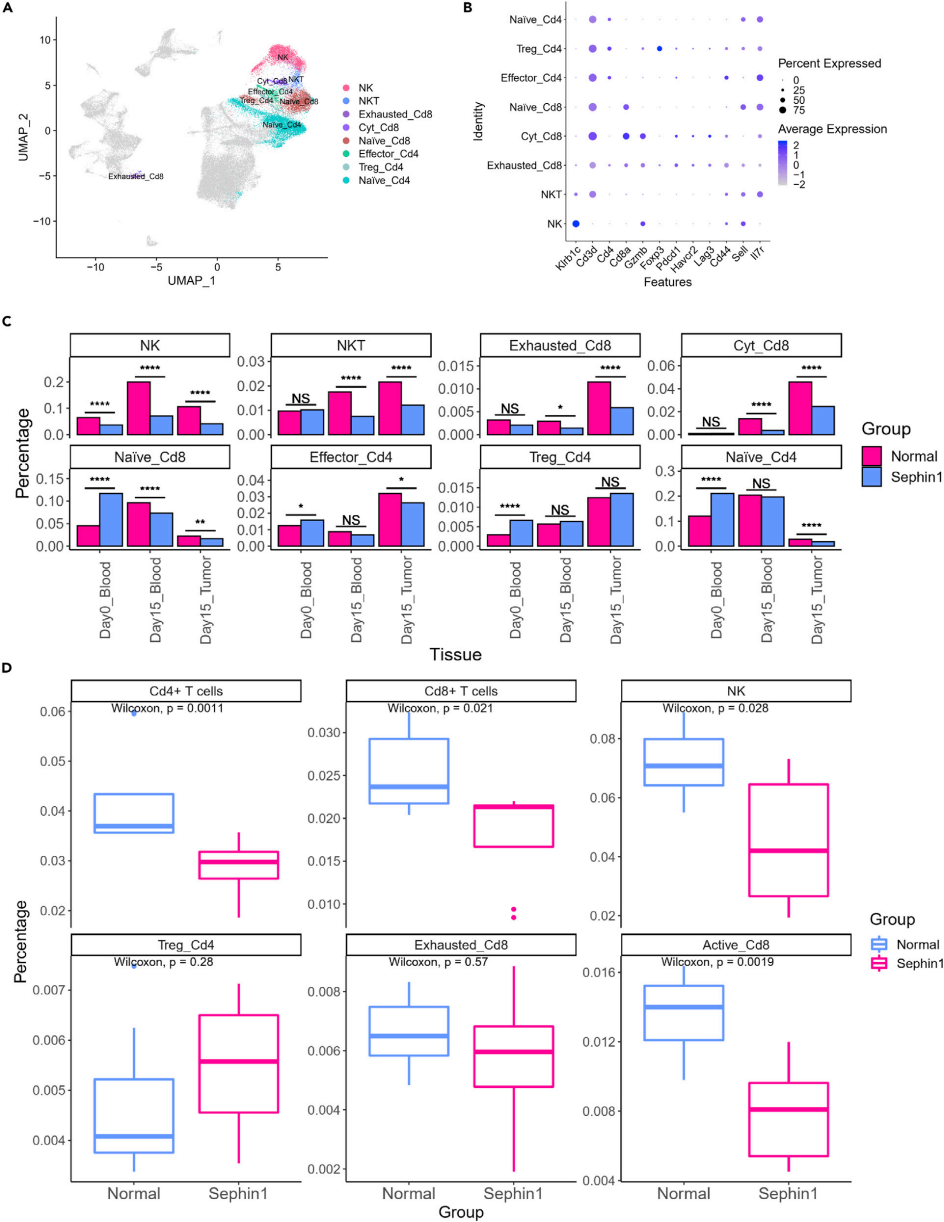
Cell distribution of lymphocytes in different sample types (A) A UMAP plot showing the detailed cell type annotation of lymphocytes NK—NK cells. NKT—NKT cells. Exhausted_Cd8—exhausted Cd8+ T cells. Cyt_Cd8—cytotoxic Cd8+ T cells. Naive_Cd8—naive Cd8+ T cells. Effector_Cd4—effector Cd4+ T cells. Treg_Cd4—regulatory T cells. Naive_Cd4—naive Cd4+ T cells. (B) Gene markers of different types of lymphocytes. Klrb1c—NK cells. Cd3d—T cells. Cd4—Cd4+ T cells. Cd8a—Cd8+ T cells. Gzmb—cytotoxic T cells. Foxp3—regulatory T cells. Pdcd1, Havcr2, Lag3—exhausted T cells. Cd44-Sell(Cd62L)+--naïve T cells. Cd44 + Sell(Cd62L)---effector T cells. (C) Distribution of lymphocyte types. The percentages of NK cells and exhausted Cd8+ T cells in the Sephin1 group were significantly reduced compared with those in the normal group. The percentages of NKT cells and cytotoxic Cd8+ T cells were significantly reduced in the blood and tumor tissue on day 15 but not in the blood on day 0. Chi-squared test was used to evaluate the distribution of normal/Sephin1 groups. ∗: p < 0.05; ∗∗: p < 0.01; ∗∗∗: p < 0.001; ∗∗∗∗: p < 0.0001. (D) Percentage changes of Cd4+ T cells, Cd8+ T cells, NK cells, regulatory T cells, exhausted Cd8+ T cells, and activated Cd8+ T cells in all immune cells determined by FACS (n = 8). Wilcoxon test was used in each cell type without adjustment, and p values were marked in the graph.

Considering the functional diversity of immune cells, we wondered which lymphocyte type plays the most crucial role in Sephin1-induced immunosuppression. We focused on several key antitumor immune cell types and analyzed their transcriptional profiles. B cells showed a similar distribution and expression patterns between the normal and Sephin1 groups and thus were excluded from further analysis. The remaining four lymphocyte cell types were further annotated as 8 subtypes. In contrast to NK cells and NKT cells, Cd4+ T cells were annotated into three subgroups: effector T cells, regulatory T cells, and naive T cells. In addition, Cd8+ T cells were annotated into three subgroups: exhausted T cells, cytotoxic T cells, and naive T cells (Figure 2A). Different markers were used for cell annotation (Figure 2B). Compared with the blood samples collected on day 0, the blood and tumor samples collected on day 15 showed more remarkable alterations in immune cell compositions induced by Sephin1 (Figure 2C). The percentages of NK cells, NKT cells, exhausted Cd8+ T cells, and cytotoxic Cd8+ T cells were all reduced significantly in the blood and tumor samples collected on day 15 from the Sephin1 group. However, the change was not significant in the day 0 blood samples. The percentage of naive_Cd8+ T cells was slightly reduced in day 15 samples but increased in day 0 samples. The number of regulatory T cells was slightly increased in day 15 samples but not as high as in day 0 samples. Fluorescence-activated cell sorting (FACS) was used to verify these results, and the percentage of antitumor-related immune cell types in all immune cells was calculated and compared between the normal and Sephin1 group in the tumor microenvironment. The percentages of Cd4+ and Cd8+ T cells, NK cells, and active Cd8+ T cells were all significantly downregulated in the Sephin1 group, while the regular Cd4+ T cells and exhausted Cd8+ T cells did not vary significantly between the two groups (Figures 2D and S4A–S4G). We observed that in the FACS analysis results, the percentages of exhausted and activated Cd8+ T cells and NK cells were significantly reduced, but the percentage of regulatory T cells was increased.

**Figure 3:**
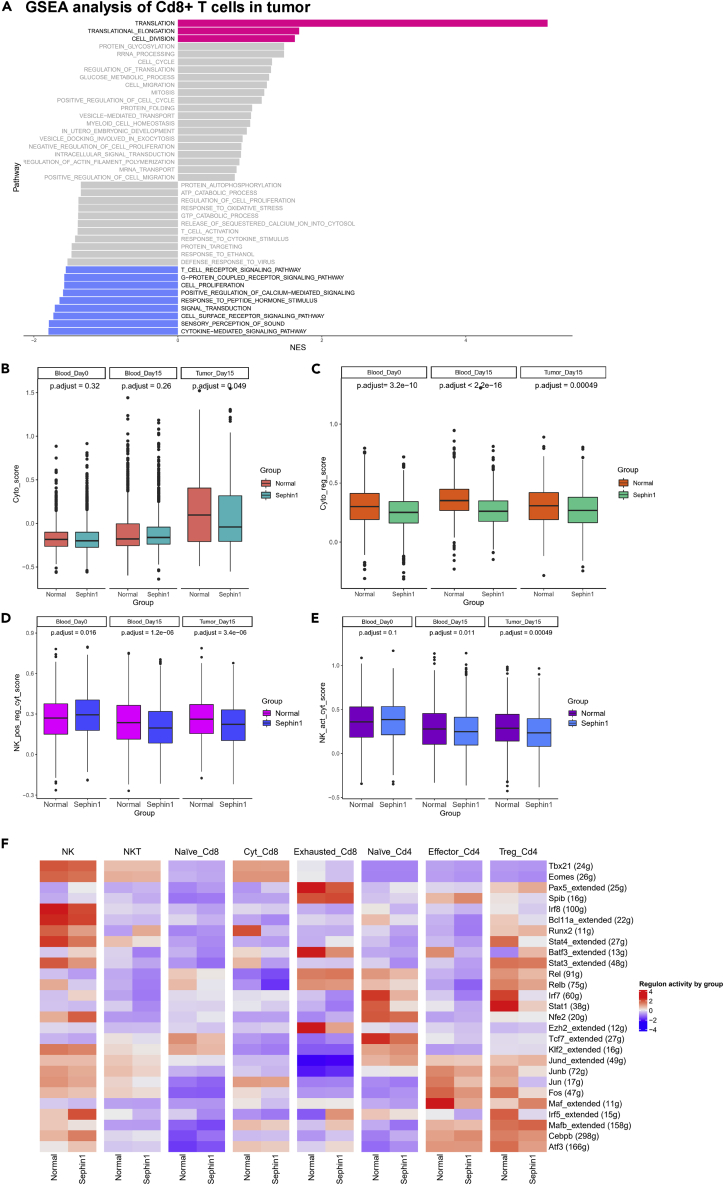
Changes in expression levels in Cd8+ T cells and NK cells (A) GSEA of Cd8+ T cells in tumor tissue. Right: pathways that were upregulated in the Sephin1 group. Left: pathways that were downregulated in the Sephin1 group. Pink/blue: significant up/downregulated pathways in the Sephin1 group. Gray: nonsignificant pathways in the Sephin1 group. (B) Expression scores for cytotoxicity-related genes in Cd8+ T cells. The expression score was significantly lower in the tumor tissue in the Sephin1 group. The p values were calculated by Wilcoxon test and adjusted by Bonferroni-Holm method. (C) Expression scores for genes related to the positive regulation of Cd8+ cell cytotoxicity. Expression scores were significantly downregulated in the Sephin1 group in the blood samples collected on day 0 and day 15 and tumor tissues. The p values were calculated by Wilcoxon test and adjusted by Bonferroni-Holm method. (D) Expression scores for genes related to the positive regulation of NK-cell activity. In the blood and tumor tissue samples collected on day 15, all the expression scores in the Sephin1 group were significantly lower than those in the normal group. The p values were calculated by Wilcoxon test and adjusted by Bonferroni-Holm method. (E) Expression scores for genes related to NK-cell activity. For the blood and tumor tissue samples collected on day 15, the scores were significantly lower in the Sephin1 group. The p values were calculated by Wilcoxon test and adjusted by Bonferroni-Holm method. (F) SCENIC analysis of different lymphocyte subtypes between normal and Sephin1 group. The activity of the Atf3 regulon was upregulated in exhausted Cd8+ T cells, cytotoxic Cd8+ T cells, NK cells, and NKT cells but downregulated in regulatory T cells.

Apart from the composition change, Sephin1 also affected the expression levels in lymphocytes. To determine the effect of Sephin1 on the expression patterns of different lymphocyte subtypes, we analyzed differential expression levels between the normal and Sephin1 groups. In Cd8+ T cells, we found that several important pathways related to antitumor immunity were significantly downregulated in the Sephin1 group (Figure 3A). The significantly downregulated pathways included cytokine-mediated signaling, cell surface receptor signaling, signal transduction, response to peptide hormone stimulus, positive regulation of calcium-mediated signaling, cell proliferation, G-protein-coupled receptor signaling, and the T cell receptor signaling pathway. The downregulation of these pathways indicated that the function of Cd8+ T cells was suppressed significantly. The significantly upregulated pathways in the Sephin1 group included pathways related to translation, translational elongation, and cell division, which indicated the loss of feedback regulatory function in the translational process.

To identify the specific pathways most affected by Sephin1, we calculated the expression score of Cd8+ T cells using the Seurat package AddModuleScore. Genes involved in T cell cytotoxicity and positive regulation of T cell cytotoxicity from Gene Ontology were analyzed. Genes involved in T cell cytotoxicity had significantly lower expression scores in the day 15 tumor microenvironment samples but not the day 0 or day 15 blood samples from the Sephin1 group (Figure 3B). However, the expression levels of genes related to the positive regulation of T cell cytotoxicity were significantly downregulated in all three sample types (Figure 3C). These results showed that the activity of Cd8+ T cells was inhibited by Sephin1, especially in the tumor microenvironment. *In vitro* analysis of Cd8+ T cells comparing the control and Sephin1 groups also showed that the percentage of cells expressing interferon-gamma (IFN-γ) in the Sephin1 group was significantly lower than that in the control group, which indicated that the Sephin1 group had lower Cd8+ T cell activity *in vitro* (Figures S5A and S5B).

The scores of genes related to NK-cell positive regulation (Figure 3D) and activity (Figure 3E) from Gene Ontology were also calculated, and they exhibited similar patterns. Gene set enrichment analysis (GSEA) of NK cells also demonstrated that genes related to NK-cell activity, including genes involved in the cytokine-mediated signaling pathway and induction of apoptosis, were downregulated in the tumor Sephin1 group (Figure S3D). The Treg differentiation score was also calculated for regulatory T cells, and the score was significantly upregulated in PBMCs in the Sephin1 group but downregulated in tumor samples (Figure S3C). The gene expression levels of these gene sets were also calculated and compared among different clusters in different sample types (Figure S3A). The expression levels of NK-cell positive regulation- and activity-related genes were downregulated in both the blood and tumor microenvironment on day 15 but slightly upregulated in the blood on day 0. We also performed SCENIC analysis of all lymphocyte subtypes (Figure 3F). The activity of the Atf3 regulon was upregulated in exhausted Cd8+ T cells, cytotoxic Cd8+ T cells, NK cells, and NKT cells but downregulated in regulatory T cells. These results indicated that the inhibitory effect of Sephin1 might vary among different cell types but have similar immunoinhibitory functions. The PI3K-related regulons showed similar patterns in these cell types, and the activity scores of PI3K-related regulons were all downregulated in both cytotoxic T cells and regulatory T cells.

### TCR analysis revealed that the clonal expansion of specific T cell subtypes is inhibited by Sephin1

By analyzing carboxyfluorescein diacetate succinimidyl ester (CFSE)-stained Cd8+ T cells *in vitro*, we found that the Sephin1 group had a significantly lower proliferative ability than the control group (Figures S5A and S5B). Although the overall expression of proliferation-related genes was not different between the two groups, the clonal expansion of T cells in tumor was inhibited in the Sephin1 group based on single-cell T cell receptor (TCR) analysis. TCRs were separated into four types: hyperexpanded, large, medium, and small based on the described methods.

**Figure 4:**
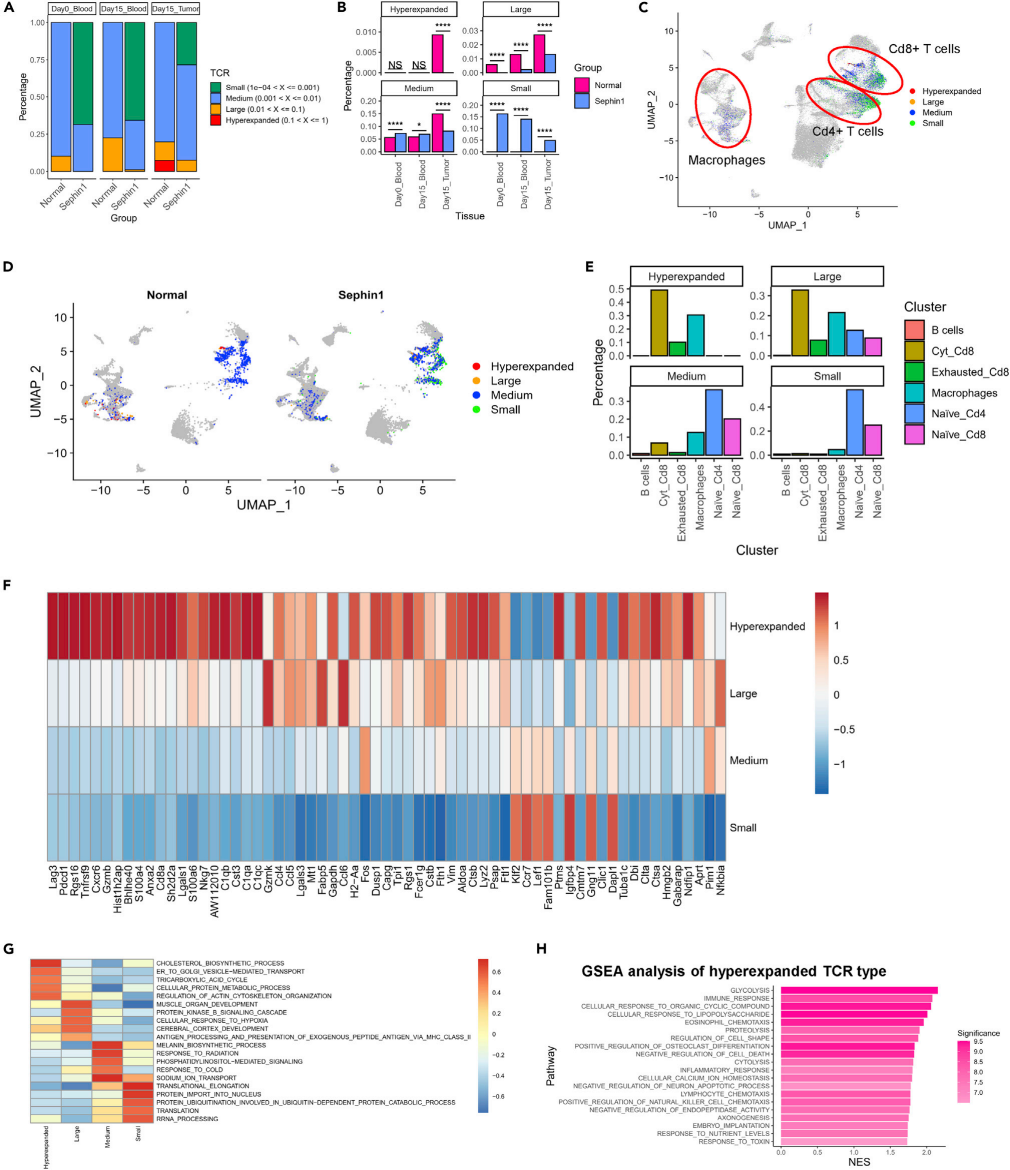
Analysis of the distributions and expression patterns revealed by TCR enrichment sequencing results (A) Distribution of different TCR clonotypes based on clonotype frequency: hyperexpanded, large, medium, and small (from high to low). Clonotypes in the Sephin1 group tended to have a lower frequency. (B) Distribution of cells belonging to different TCR types. The percentage of cells with a higher TCR frequency, including the hyperexpanded and large TCR types, was significantly lower in the Sephin1 group for all three sample types. Chi-squared test was used to evaluate the distribution of normal/Sephin1 groups. ∗: p < 0.05; ∗∗: p < 0.01; ∗∗∗: p < 0.001; ∗∗∗∗: p < 0.0001. (C) UMAP plot of the distribution of all four TCR types. (D) UMAP plot of the TCR types split by group. All the cells belonging to the hyperexpanded TCR type were in the normal group. (E) Distribution of the four TCR types in different cell types. In addition to T cells, macrophages also exhibited populations in the hyperexpanded, large, and small types. (F) Genes specifically expressed in different TCR types. The hyperexpanded type had higher expression activity, and the expression levels of cytotoxicity-related genes, such as Gzmb and Gzmk, were also significantly higher. (G) GSVA of all four TCR types. Enrichment analysis was performed based on the biological process database for GO. The top 5 most highly enriched pathways for each cell type are displayed. (H) GSEA of the hyperexpanded type. NES—normalized enrichment score. Significance was calculated as –log_10_(P).

First, we calculated the percentages of different categories of clonotypes in one sample (Figure 4A). We found that for all three sample types, the samples in the Sephin1 group had a lower percentage of highly expanded clonotypes. In the blood samples collected on day 0 or day 15, the percentages of TCR types in the large and medium categories were significantly lower in the Sephin1 group. In the day 15 tumor samples, the hyperexpanded, large, and medium TCR types were also significantly downregulated in the Sephin1 samples. Apart from the clonotype percentages, the percentage of cells belonging to each TCR type showed a similar pattern. The percentages of cells belonging to the hyperexpanded and large types were downregulated in the Sephin1 samples for all three sample types, while the percentage of the small type was upregulated (Figure 4B). The ranking of different TCR clonotypes showed similar results. Clonotypes were ranked according to their clone number proportion within the complete TCR repertoire of one sample, and the percentages of clonotypes with higher ranks were decreased in all three sample types (Figure S6B).

Further analysis found that although TCR + cells were mainly distributed in T cells, there was also a large number of TCR + cells found in the macrophage population, and a comparatively high proportion of macrophages had a hyperexpanded- or large-clonal TCR types (Figures 4 C and 4E). In addition, the percentage of highly expanded TCR types, namely, a hyperexpanded or large TCR type, was also higher in the normal group than in the Sephin1 group for both T cells and macrophages (Figure 4, Figure 6B, 4D, and 6F).

To determine the expression patterns of different TCR types, especially highly expanded TCR types, we analyzed the differentially expressed genes in different TCR types. The hyperexpanded type had much higher expression levels of cytotoxicity-related genes, such as Gzmb and Gzmk (Figure 4F). GSVA was performed according to the expression levels of the differentially expressed genes of each TCR type, and the results showed that the highly expanded TCR cell types had higher metabolic activities. Pathways such as the cholesterol biosynthetic process and tricarboxylic acid cycle were found to be highly expressed in the hyperexpanded type. Immune-related pathways, such as the cellular response to hypoxia pathway and the antigen processing and presentation of an exogenous peptide antigen via major histocompatibility complex (MHC) class II pathway, were activated in the large type (Figure 4G). Immunity-related and cell-killing-related pathways were also upregulated in the hyperexpanded type, such as the inflammatory response and positive regulation of NK cell chemotaxis pathways (Figure 4H).

### Macrophages in the Sephin1 group are more likely to be in an M2-polarized state

**Figure 5:**
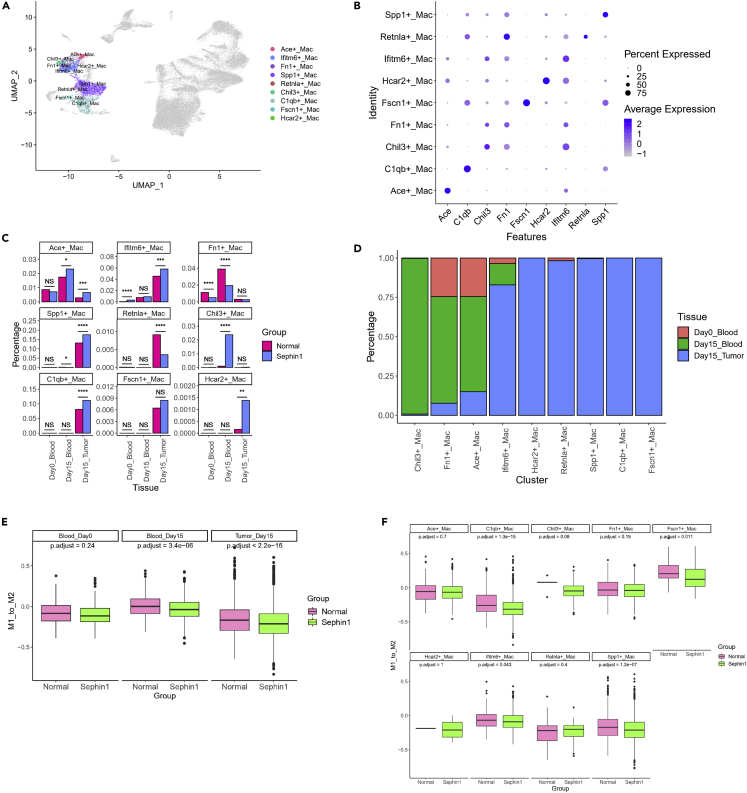
Distribution and expression patterns of macrophages (A) Subtypes of macrophages named by the specifically highly expressed genes of each cluster. Nine subtypes were identified. (B) Gene markers of each macrophage subtype. (C) Distribution of macrophage subtypes. Chil3+ and Hcar2+ macrophages mainly existed in the Sephin1 group. Chi-squared test was used to evaluate the distribution of normal/Sephin1 groups. ∗: p < 0.05; ∗∗: p < 0.01; ∗∗∗: p < 0.001; ∗∗∗∗: p < 0.0001. (D) Distribution of macrophage subtypes in different tissues. Chil3+, Fn1+ and Ace + macrophages mainly existed in the blood, and the other subtypes mainly existed in tumor tissues. (E) Characteristics of the M1-M2 polarization pattern of all macrophages. The M1_to_M2 score was calculated by subtracting the M2 expression score from the M1 expression score. A higher M1_to_M2 score indicated that the cells tended to exhibit M1 polarization. Macrophages in the blood and tumor tissues collected on day 15 in the Sephin1 group tended to exhibit M2 polarization over M1 polarization. The p values were calculated by Wilcoxon test and adjusted by Bonferroni-Holm method. (F) M1_to_M2 scores of macrophage subtypes. Subtypes that mainly existed in the blood, including Ace+, Chil3+, and Fn1+ macrophages, were not significantly different between the normal and Sephin1 groups. Subtypes in tumor tissues tended to exhibit M2 polarization in the Sephin1 group, except for Retnla+ and Hcar2+ macrophages, which each contained a small number of cells. The p values were calculated by Wilcoxon test and adjusted by Bonferroni-Holm method.

In addition to antitumor lymphocytes, macrophages also play key roles in the tumor microenvironment. Thus, we also analyzed the characteristics and functions of macrophages in different samples. Macrophages in all merged samples were divided into 11 clusters with Seurat (Figure S7A), and we annotated them into 9 subtypes named based on their specific marker genes (Figures 5A and 5B). Chil3+, Fn1+, and Ace + macrophages mainly existed in the blood. Ifitm6+, Hcar2+, Retnla+, Spp1+, C1qb+, and Fscn1+ macrophages mainly existed in tumor tissues (Figure 5D). There were two macrophage subgroups that mainly existed in the Sephin1 group: Chil3+ macrophages, which mainly existed in the day 15 blood samples, and Hcar2+ macrophages, which mainly existed in day 15 tumor samples (Figure 5C). GSVA of different macrophage subtypes showed that the highly expressed genes in the Chil3+ group were enriched in the regulation of G-protein-coupled receptor protein signaling and negative regulation of nuclear factor κB (NF-κB) pathways (Figures S7C and S8A).

There are two macrophage polarization states, M1 and M2. Typically, M1 macrophages produce type I proinflammatory cytokines and have antitumorigenic functions, while M2 macrophages produce type II cytokines and have protumorigenic functions.^52^^,^^53^ We then analyzed the expression levels of genes related to the M1- and M2-polarized states^54^ by calculating the gene expression scores for M1 and M2 polarization with AddModuleScore followed by the M1_to_M2 score determined by subtracting the M2 score from the M1 score. The higher the M1_to_M2 score was, the more the cells were polarized toward the M1 state. We found that in the blood and tumor tissues collected on day 15, the macrophages in the Sephin1 group were significantly polarized toward the M2 state compared with those in the normal group, but the variation in the day 0 blood samples was not significant (Figures 5E and S7B). We then analyzed the M1- and M2-polarized states of different macrophage subtypes. Except for two subtypes with too few cells (Retnla+ macrophages and Hcar2+ macrophages), we found that the subtypes mainly existing in tumor tissues were more tended to M2 polarization, including the Spp1+, Clqb+, Ifitm6+, and Facn1+ macrophages (Figure 5F).

GSEA was also performed on macrophages in tumor tissue to compare the Sephin1 and normal groups. We selected the 20 most upregulated and downregulated pathways between the two groups based on the normalized enrichment score (NES) (Figure S7D). We found that pathways related to T cell activities and antigen processing and presentation were downregulated in the Sephin1 group. In contrast, the pathway related to the ISR process such as the cellular response to hypoxia pathway was upregulated in the Sephin1 group. In addition, SCENIC analysis of macrophages also indicated that the ISR-related regulon, i.e., the Atf3 regulon, had higher activity in the Sephin1 group (Figure S8C).

### The macrophage subtype with TCR expression may have important functions in antitumor immunity

**Figure 6:**
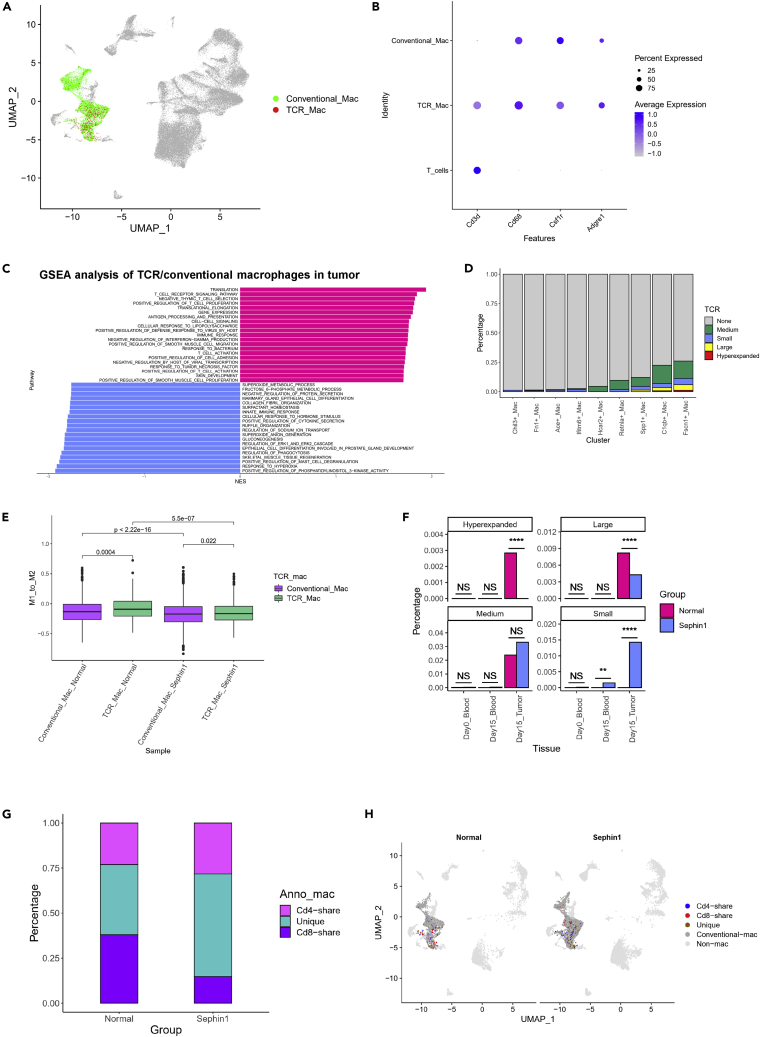
Characteristics of TCR + macrophages (A) Distribution of TCR + macrophages (TCR_Mac) and other conventional macrophages (Conventional_Mac). (B) Expression of gene markers of T cells and macrophages in TCR + macrophages. (C) GSEA of differentially expressed genes between TCR + macrophages and conventional macrophages in tumors. Upregulated genes were enriched in pathways related to T cell activities. (D) Distribution of different TCR types in macrophage subtypes. TCR + macrophages were mostly enriched in Fscn1+ macrophages. (E) M1_to_M2 scores of conventional macrophages and TCR + macrophages in tumor samples. TCR + macrophages tended to be more M1 polarized than conventional macrophages in the normal group and were more significantly affected by Sephin1. The p values were calculated by Wilcoxon test and adjusted by Bonferroni-Holm method. (F) Distribution of different TCR types in macrophages. The percentages of highly proliferated TCR types, i.e., the hyperexpanded and large TCR types, were downregulated in the Sephin1 group. Chi-squared test was used to evaluate the distribution of normal/Sephin1 groups. ∗: p < 0.05; ∗∗: p < 0.01; ∗∗∗: p < 0.001; ∗∗∗∗: p < 0.0001. (G and H) Distribution of macrophages which had shared TCRs with T cells in tumor microenvironment. Macrophages in the Sephin1 group had lower percentage of shared TCRs with Cd8+ T cells.

By TCR analysis, we found that TCRs existed not only in T cells but also in macrophages and that the percentage of TCR + macrophages for all three TCR types was as high as approximately 0.3 (hyperexpanded type, Figure 4E). In addition, GSEA comparing macrophages between the normal and Sephin1 groups indicated that pathways related to T cell activities were affected by Sephin1 (Figure S7D). These results indicated that macrophages with TCR sequences might have important functions in the immune system and antitumor procedures. Research on CD3^+^ macrophages has found that this cell type can produce proinflammatory cytokines,^55^ yet their role in antitumor immunity is still unknown. In our research, we found that Cd3+ macrophages, especially TCR + macrophages, may had important functions in antitumor immune activities. TCR + macrophages existed in both the blood and tumor tissues but were more enriched in the tumor microenvironment (Figures 6A and S8E). Approximately 13.7% of the macrophages in tumors were TCR+, but only approximately 0.5% of the macrophages in the blood were TCR+. Marker genes for both macrophages and T cells were highly expressed by this cell type, including Cd3d for T cells and Cd68, Csf1r, and Adgre1 for macrophages (Figure 6B). GSEA comparing TCR + macrophages and conventional macrophages indicated that the genes upregulated in TCR + macrophages were more enriched in pathways related to T cell activation and regulation and that the downregulated genes were enriched in pathways related to the innate immune response, cytokine secretion, and other related pathways (Figure 6C). The hyperexpanded and large TCR types mainly existed in C1qb+ and especially Fscn1+ macrophages, which mainly existed in tumor tissues (Figure 6D). GSVA of TCR + macrophages was also performed by TCR type (Figure S8D). In the hyperexpanded macrophage group, the most upregulated genes were enriched in the positive regulation of the T cell-mediated cytotoxicity pathway, which was similar to cytotoxic T cell functions. This result also indicated that TCR + macrophages might perform antitumor functions in both macrophage-like and T cell-like ways.

We also calculated the M1_to_M2 scores of both conventional macrophages and TCR + macrophages with AddModuleScore (Figure 6E). TCR + macrophages had significantly higher M1_to_M2 scores in the normal group, implying that these macrophages were more likely to have antitumor functions. The differences between the two macrophage types were less significant in the Sephin1 group, but both cell types were more polarized toward the M2 state in the Sephin1 group. These results showed that TCR + macrophages were likely to have antitumor functions mediated through both macrophage- and T cell-related pathways and that these functions could be inhibited by Sephin1.

Distribution analysis of the different types of TCR + macrophages in different tissue types showed that the percentages of the hyperexpanded and large types of macrophages were significantly downregulated in the Sephin1 group, while the percentages of the medium and small types of macrophages were upregulated, which was consistent with the overall TCR + cell patterns (Figure 6F). In addition, we also calculated the percentage of TCR + macrophages with shared TCR sequences with Cd4+ (Cd4-share) and Cd8+ T cells (Cd8-share) in the tumor tissue. It turns out that in the Sephin1 group, the Cd8-share macrophages were significantly fewer than those in the normal group (Figures 6G and 6H), which indicated that the TCR + macrophages might have got the TCR sequences through interaction with T cells. The existence of TCR + macrophages was also proved by FACS results in the tumor microenvironment, and there was no significant difference between the normal and Sephin1 group (Figures S9A–S9C). In addition, we also found that TCR + macrophages existed in the normal mouse spleen tissue (Figures S9D and S9E).

### Sephin1 suppresses antitumor immunity in cell-cell communication level

**Figure 7:**
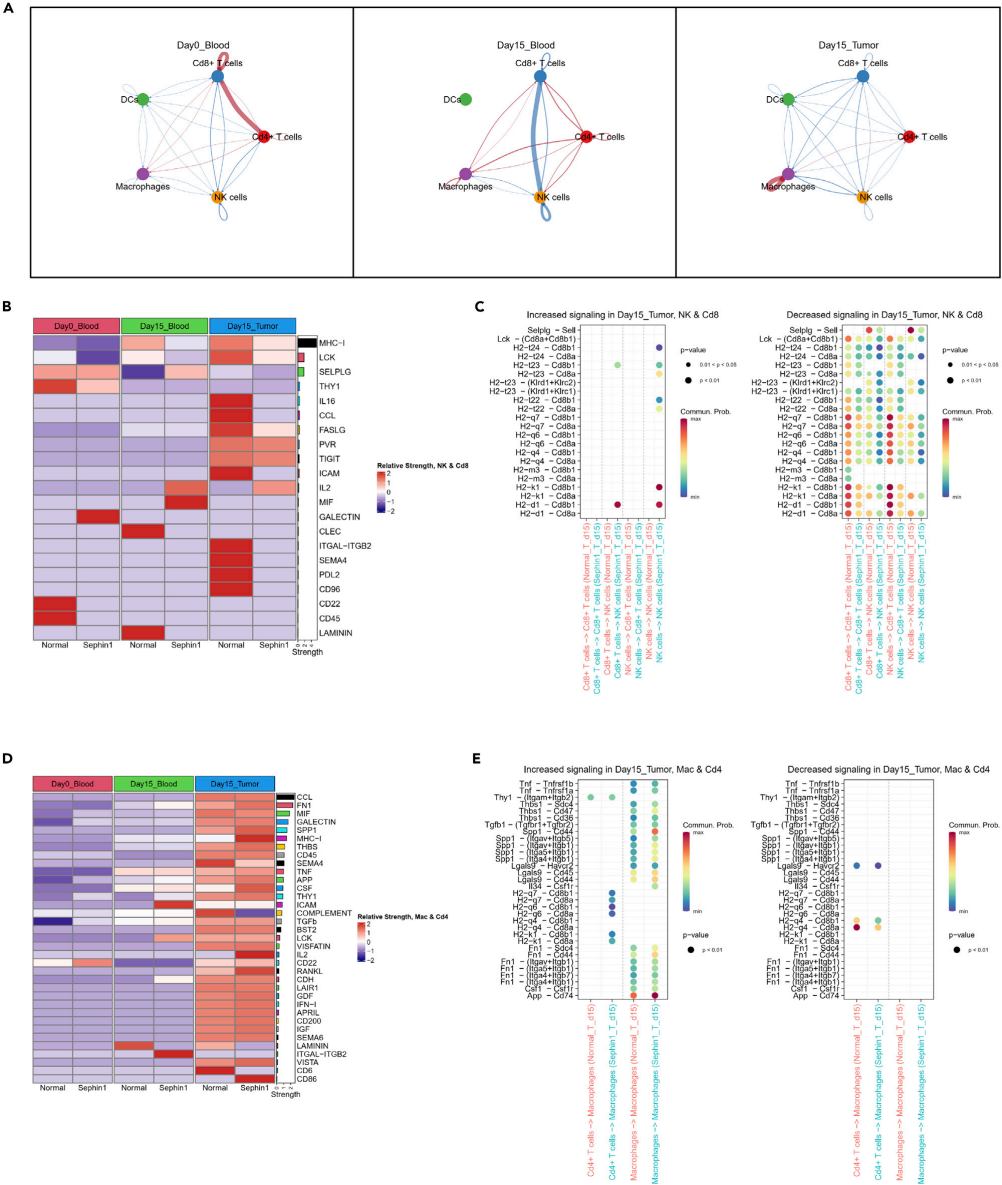
Cell-cell communication analysis of selected cell types (A) Differentiated communication strengths between the normal and Sephin1 groups of different tissues. Red: upregulated in the Sephin1 group. Blue: downregulated in the Sephin1 group. All three tissue types had the downregulation tendency in the Sephin1 group, especially in tumor. (B) Mostly differentiated communication pathways between Cd8+ T cells and NK cells, which were downregulated in the Sephin1 group of all three tissue types, including communications of NK-NK, Cd8-Cd8, NK-Cd8, and Cd8-NK. (C) Ligand-receptor pairs of mostly downregulated pathways in the tumor tissue, including MHC-I, LCK, and SELPLG pathways. (D) Mostly differentiated communication pathways between Cd4+ T cells and macrophages, which were upregulated in the Sephin1 group of all three tissue types. (E) Ligand-receptor pairs of mostly upregulated pathways in the tumor tissue, including FN1, GALECTIN, SPP1, MHC-I, THBS, TGFb, APP, THY1, TNF, and CSF pathways.

In order to find the differentially expressed genes and communication strengths between the normal and Sephin1 group, we calculated the cell-cell communication score and strength between different samples by CellChat.^56^ In the tumor tissue, most communication strengths were downregulated in the Sephin1 group, except the communications of macrophages-macrophages and macrophages-Cd4+ T cells. In all three tissue types, communications between Cd8+ T cells and NK cells were all downregulated in the Sephin1 group, which indicated that these cell-cell communications may have more important functions (Figure 7A).

By analyzing the differentially expressed ligand-receptor pairs between the normal and Sephin1 groups between Cd8+ T cells and NK cells, we found that these pairs were mostly enriched in MHC-I related pathways (Figure 7B). Besides, the strengths of ligand-receptor pairs in MHC-I pathway were all downregulated in the Sephin1 group of all three tissue types. This result indicated that the influence of Sephin1 to the antitumor immunity was enriched in MHC-I related pathways. Apart from this, two other pathways with relatively high overall strength also showed reduced tendency in the Sephin1 group, including lymphocyte cell-specific protein-tyrosine kinase (LCK) pathway and selectin P ligand (SELPLG) pathway (Figure 7B). In the tumor microenvironment, Selplg-Sell and Lck-(Cd8a + Cd8b1) pairs were included in the significantly decreased pairs in Sephin1 group compared to normal (Figure 7C).

On the contrary, the communication strength of macrophages-Cd4+ T cells and macrophages-macrophages was upregulated in the Sephin1 group. Therefore, we also analyzed the differentially expressed communication pathways between these two cell types (Figure 7D). Pathways with high expression levels and also significantly upregulated in the Sephin1 group included fibronectin 1 (FN1), GALECTIN, secreted phosphoprotein 1 (SPP1), MHC-I, thrombospondin 1 (THBS), TGFb, amyloid beta precursor protein (APP), Thy-1 cell surface antigen (THY1), tumor necrosis factor (TNF), and colony stimulating factor (CSF), in which many were related to antitumor immunity suppression. We further analyzed the ligand-receptor pairs in these pathways (Figures 7E and S10B). Although MHC-I pathway was upregulated in the Sephin1 group of tumor tissue by overall strength (Figure 7D), the number of upregulated ligand-receptor pairs was less than downregulated ones (Figure 7E). In addition, most upregulated pairs were enriched in the macrophage-macrophage interaction, which was also verified by immunofluorescence results (FN1-CD44 & SPP1-CD44 pairs. Figures S11A–S11F). Furthermore, in the tumor tissue, the upregulated ligand-receptor pairs mostly existed in the macrophage-macrophage communication including Thbs1-, Spp1-, Lgals9-, and Csf-related pairs, which were highly related to immunity suppression.

## Discussion

Sephin1 is a selective inhibitor of PPP1R15A and can inhibit dephosphorylation of eIF2α by inhibiting the formulation of the PPP1R15A-PP1c complex.^46^ eIF2α is a key component of the integrated stress response process (ISR), which can be induced by both extrinsic factors and intrinsic cellular stresses, including oncogene activation.^16^^,^^57^^,^^58^ Usage of Sephin1 in mammals can lead to a promotion of ISR activity; thus, it is used as a potential treatment in neuron motor-, and proteostasis-related diseases.^48^^,^^49^^,^^50^ In our study, we found that the usage of Sephin1 in mice can lead to antitumor immunity suppression, which is most likely to be achieved by ISR process by single-cell expression analysis. In the C56BL/6 mice injected subcutaneously with B16F1 cells, the tumor growth rate in the Sephin1 group was significantly higher than that in the normal group, which indicated a possible relationship between the ISR process and antitumor immune activities. SCENIC analysis of the single-cell data for all immune cells between the normal and Sephin1 groups showed that all three sample types showed higher activities of the Atf3 regulon, which includes core genes related to the ISR, and other related regulons in the Sephin1 group, which indicated a higher ISR level. However, regulons related to immune cell activities were downregulated in the Sephin1 group, which indicated the induction of an immunosuppressive effect by Sephin1. To fully understand the suppressive effect on antitumor immune activities mediated by different kinds of immune cell types, we analyzed the expression and distribution patterns of different cell types in different tissues.

Lymphocytes that are important for antitumor immunity were more likely to be affected by Sephin1 injection. NK cells, NKT cells, and Cd8+ T cells were all significantly reduced among the immune cells in tumor tissue in the Sephin1 group, while regulatory T cells were more enriched. In addition, as key antitumor cell types in innate and adaptive immune systems,^59^^,^^60^ Cd8+ T cells and NK cells also exhibited lower expression and cell-killing activities in the Sephin1 group. As for NKT cells, previous studies have shown that depending on the cell type, NKT cells can either suppress (type I NKT cells) or promote (type II NKT cells) tumor development^61^^,^^62^; thus, the effects of the reduction in NKT cells may be controversial. In addition, the enrichment of regulatory T cells in the Sephin1 group also indicated suppression of antitumor immunity.^63^ SCENIC analysis also indicated that Atf3 regulon activity in the Sephin1 group in tumor tissue was higher in antitumor cell types such as NK cells, NKT cells, and Cd8+ T cells but lower in the suppressive T cell type, regulatory Cd4+ T cells,^64^ which also indicated the antitumor suppression effects of Sephin1.

By analyzing the TCR clonotype distribution, we found that tumor-specific T cell proliferation was also suppressed by Sephin1 injection. The TCR sequencing analysis indicated that highly expanded TCR clonotypes were significantly decreased in the Sephin1 group in terms of the numbers of both clonotypes and clones. Highly expanded TCR clonotypes were more enriched in cytotoxic Cd8+ T cells and macrophages and had higher expression of genes related to cytotoxicity-related pathways, which indicated that these cells were important for tumor-specific identification and cell killing. In addition, clonotypes with a lower clone number were more enriched in naive T cells.

Macrophages can also exert important antitumor immune activities. Macrophages can have a tendency to polarize toward the M1 or M2 state^65^^,^^66^ but exist along a continuum and cannot be distinctly separated into the M1 or M2 type.^54^^,^^67^ Previous studies have demonstrated that M1 macrophages are proinflammatory, while M2 macrophages are anti-inflammatory.^68^ In the tumor microenvironment, M1-like macrophages are more likely to have antitumor functions, while M2 macrophages have the opposite impact.^69^ In our experiment, by evaluating the polarization tendency through evaluation of a series of M1- and M2-related genes, we found that in the Sephin1 group, macrophages tended to exhibit an M2-polarized state, which was more likely to promote tumor development. In addition, macrophage subtypes in the tumor microenvironment were more deeply affected by Sephin1 than those in the blood, indicating that Sephin1 had stronger influence on tumor-associated macrophages.

Previous studies have shown that CD3+TCRαβ+ macrophages can produce proinflammatory cytokines and have import functions in infection-related biological process,^55^ and this kind of macrophages may be generated through the trogocytosis between macrophages and T cells.^70^^,^^71^ However, the traditional trogocytosis theory only included the exchange of membrane and membrane-associated proteins. In our study, the single-cell sequencing data were at mRNA level, which indicated that a large number of macrophages in the tumor microenvironment contained the mRNAs of TCRs and other T cell-related genes. This phenomenon indicated that the interaction between antigen-presenting cells (APCs) and T cells may not be limited to the cell surface but also involve a deeper level of substance exchange. In addition, clonotype analysis of TCR + macrophages indicated that macrophages with higher TCR frequency were more likely to be suppressed in the Sephin1 group. Besides, TCR + macrophages tended to undergo M1 polarization more than conventional macrophages and were also more enriched in the tumor microenvironment. These results all indicated that TCR + macrophages could play vital roles in both T cell- and macrophage-related pathways. The suppressive effect of Sephin1 on this cell type was also more significant than that on conventional macrophages.

Based on these results, we further analyzed the cell-cell communication between Cd8+ T cells, Cd4+ T cells, NK cells, macrophages, and DCs and analyzed cell pairs that had similar patterns between all three tissue types. In the cell-cell communications that were downregulated in the Sephin1 group, MHC-I, LCK, and SELPLG pathways were significantly downregulated and also had relatively high communication strengths. The SELPLG pathway is known with cell-cell adhesion function and may have functions in antitumor immunity.^72^^,^^73^ MHC-I and LCK pathways have important functions in antigen presenting and also associated with each other. LCK is known as inducing initial TCR-triggering event.^74^ FN1, GALECTIN, SPP1, THBS, TGFb, APP, THY1, TNF, and CSF pathways were upregulated in the Sephin1 group. Most of these pathways were antitumor suppressive. GALECTIN can lead to T cell inhibition by Lgals9-Havcr2 interaction.^75^ SPP1 can facilitate immune escape in tumor tissues.^76^ THBS1 can limit antitumor immunity by CD47-dependent regulation of innate and adaptive immune cells.^77^ CSF1/CSF1R pathway can lead to inhibition to T cell checkpoint immunity.^78^ TNF is an important pathway in cell apoptosis, which is also highly related to ISR process, and can also trigger the death signaling in immune cells.^79^ TGFb is known as an important marker of M2 macrophages, which is highly related to protumor effects.^80^ APP and THY1 pathways may also have important functions in antitumor immunity; however, research on these two pathways about antitumor immunity is still lacking.

In conclusion, the injection of Sephin1 could lead to the suppression of antitumor immunity during the development of implanted B16F1 tumors. This finding was also verified in another model using 4T1 tumor cells. As a selective inhibitor of PPP1R15A, Sephin1 can inhibit the binding of PPP1R15A to the PPP1R15A-PP1c complex and promote the integrated stress response in mice. From our results, we inferred that PPP1R15A and other ISR-related genes and their protein products could be important potential targets in tumor immunotherapy. The ISR is also an important pathway related to the immune response in mammals. A macrophage subtype was identified to be highly associated with Sephin1 treatment and to play a crucial role in antitumor immunity, suggesting a potential mechanism by which Sephin1 exerts its protumorigenic effect. Furthermore, cell-cell communication analysis also proved that the antitumor-related immunity interactions were suppressed by Sephin1 in mouse blood and tumor microenvironment. In a word, PPP1R15A and its related ISR play a key role in the immune system, especially antitumor immunity, and can be used as a target for tumor immunotherapy. The inhibitor Sephin1 also has the potential for immunity-related diseases, such as autoimmune disease.^81^^,^^82^

### Limitations of the study

In this study, we mainly concentrated on the single-cell transcriptome and immune profiling of the tumor microenvironment and PBMCs in the normal and PPP1R15A-inhibited mice. There are still some limitations in our study. Firstly, we did not perform the overexpression or activation study of PPP1R15A, and the effect of PPP1R15A activation to immune system is still unknown. Secondly, the experiment was only performed on mice and mouse cells without human data, which are more meaningful for drug discovery. Thirdly, our single-cell data only contained the transcriptome and TCR data, without (B cell receptor) BCR data. The changes in BCR complexity can also be meaningful for the study of PPP1R15A function.

## STAR★Methods

### Key resources table

**Table:** 

REAGENT or RESOURCE	SOURCE	IDENTIFIER
**Antibodies**		

PE Hamster Anti-Mouse CD3e	BD Biosciences	Cat# 553064; RRID: AB_394597
FITC anti-mouse TCR β chain Antibody	BioLegend	Cat# 109205; RRID: AB_313428
PE/Cyanine7 anti-mouse/human CD11b Antibody	BioLegend	Cat# 101215; RRID: AB_312798
PerCP anti-mouse F4/80 Antibody	BioLegend	Cat# 123125; RRID: AB_893495
PE anti-mouse CD45 Antibody	BioLegend	Cat# 103105; RRID: AB_312970
FITC anti-mouse CD45 Recombinant Antibody	BioLegend	Cat# 157607; RRID: AB_2832553
Brilliant Violet 605™ anti-mouse CD4 Antibody	BioLegend	Cat# 100548; RRID: AB_2563054
Brilliant Violet 421™ anti-mouse FOXP3 Antibody	BioLegend	Cat# 126419; RRID: AB_2565933
PerCP/Cyanine 5.5-conjugated anti-mouse CD8a antibody	BioLegend	Cat# 100733; RRID: AB_2075239
PE-conjugated anti-mouse IFN-γ antibody	BioLegend	Cat# 163503; RRID: AB_2890730
InVivoMAb anti-mouse CD3ε	BioXCell	Cat# BE0001-1-5MG; RRID: AB_1107634
InVivoMAb anti-mouse CD28	BioXCell	Cat# BE0015-1-5MG; RRID: AB_1107624
CD45 Monoclonal Antibody (30-F11), PE, eBioscience™	ThermoFisher	Cat# 12-0451-83; RRID: AB_465669
CD8a Monoclonal Antibody (53-6.7), PE-Cyanine7, eBioscience™	ThermoFisher	Cat# 25-0081-81; RRID: AB_469583
NK1.1 Monoclonal Antibody (PK136), eFluor™ 450, eBioscience™	ThermoFisher	Cat# 48-5941-80; RRID: AB_2043878
CD279 (PD-1) Monoclonal Antibody (J43), APC, eBioscience™	ThermoFisher	Cat# 17-9985-82; RRID: AB_11149358

**Chemicals, peptides, and recombinant proteins**		

Ficoll-Paque PREMIUM	Amersham/GE	17544602
Sephin1	APExBIO	A8708-50
FBS	Biological Industries	04-001-1A
RPMI 1640 medium	Gibco	11875093
Pen Strep	Gibco	15140122
MACS® SmartStrainers (30μm), 4x 25pcs	Miltenyi/MACS	130-110-915
AO/PI	Nexcelom Bioscience	CS2-0106-5mL
Propidium iodide solution	Nexcelom Bioscience	CS1-0109-5mL
IL2, mouse	Novoprotein	CK24
IL7, mouse	Novoprotein	CC73
ACK Lysing Buffer	ThermoFisher	A1049201

**Critical commercial assays**		

Chromium Next GEM Single Cell 5’ Library & Gel Bead Kit v1.1	10X Genomics	PN-1000165
Chromium Single Cell 5’ Library Construction Kit	10X Genomics	PN-1000020
Chromium Single Cell V(D)J Enrichment Kit	10X Genomics	PN-1000071
Chromium Next GEM Chip G Single Cell Kit	10X Genomics	PN-1000120
Single Index Kit T Set A	10X Genomics	PN-1000213
BD Cytofix/Cytoperm™ Fixation/Permeablization Kit	BD Biosciences	554714
Cell Activation Cocktail with Brefeldin A	BioLegend	423303
MojoSort™ Mouse CD8 T Cell Isolation Kit	BioLegend	480035
CFSE Cell Division Tracker Kit	BioLegend	423801
Tumor Dissociation Kit, mouse	Miltenyi/MACS	130-096-730
LIVE/DEAD™ Fixable Violet Dead Cell Stain Kit, for 405 nm excitation	ThermoFisher	L34963
LIVE/DEAD™ Fixable Near IR (780) Viability Kit, for 633 nm excitation	ThermoFisher	L34994

**Deposited data**		

Raw and analyzed data	This paper	GEO: GSE220656

**Experimental models: Organisms/strains**		

C57BL/6Slac,mus musculus	SLAC LABORATORY ANIMAL	NA

**Software and algorithms**		

CellRanger	10X Genomics	Version 6.0.0
Seurat	https://satijalab.org/seurat/index.html	Version 3.2.3
scRepertoire	http://www.bioconductor.org/packages/release/bioc/html/scRepertoire.html	Version 1.2.1
Python	https://www.python.org/	Version 2.7.5
R	https://cran.r-project.org/	Version 3.3.5
SCENIC	https://github.com/aertslab/SCENIC	Version 1.1.2-01
GENIE3	http://www.bioconductor.org/packages/release/bioc/html/GENIE3.html	Version 1.8.0
GSVA	http://www.bioconductor.org/packages/release/bioc/html/GSVA.html	Version 1.30.0
Fgsea	http://www.bioconductor.org/packages/release/bioc/html/fgsea.html	Version 1.8.0
ggplot2	https://cran.r-project.org/	Version 3.3.5
Ggpubr	https://cran.r-project.org/	Version 0.4.0
Pheatmap	https://cran.r-project.org/	Version 1.0.12
ComplexHeatmap	https://cran.r-project.org/	Version 2.8.0
CellChat	https://github.com/sqjin/CellChat	Version 1.0.0

**Other**		

Original code	This paper	https://github.com/LisaWang2022/B16F1-mouse-single-cell-analysis

### Resource availability

#### Lead contact

Further information and requests for resources and reagents should be directed to and will be fulfilled by the lead contact, Xiangyin Kong (xykong@sibs.ac.cn).

#### Materials availability

This study did not generate new unique reagents.

### Experimental model and subject details

#### Mice

The mouse models used in this study were obtained from SLAC LABORATORY ANIMAL. The strain was C57BL/6Slac. 6-8 weeks male mice were used in this study, and they were separated into normal and Sephin1 groups randomly. Animal study protocols were approved by the Institutional Animal Care and Use Committee of Shanghai Institute of Nutrition and Health, CAS. All animals were housed under specific pathogen-free (SPF) conditions according to the National Institutes of Health’s Guide for the Care and Use of Laboratory Animals.

### Method details

#### Reagent preparation

A Sephin1 solution was prepared before injection. Sephin1 (50 mg, APExBIO, A8708-50) was first dissolved in 625 μl DMSO and then dissolved in 12.5 ml PBS. The final solution contained 4 mg/ml Sephin1 and 5% DMSO. This solution was used for mouse injection. The same volume of DMSO solution with the same percentage (5% DMSO in PBS) was used to treat the control group.

#### Mouse models

Six-to eight-week-old male C57BL/6 mice were used in this study. Mice were first separated into two groups, the normal group and the Sephin1 group (n=10). The Sephin1 group was injected intraperitoneally with 100 μl Sephin1 solution prepared as described above, and the normal group was injected with an equal 5% DMSO-PBS solution. Both groups were injected three times per week for two weeks. Two days after the last injection, two mice from each group were randomly selected, and their peripheral blood mononuclear cells (PBMCs) were harvested for single-cell sequencing; the remaining mice were subcutaneously inoculated with $3\times 10^{5}$ B16F1 cells. The shortest and longest tumor diameters (d and D) were measured with a Vernier caliper every one or two days for two weeks, and tumor volume (V) was calculated as: $V=d^{2}\times D/2$. The tumor tissues were collected after two weeks of development for further experiments.

In addition to studying B16F1 tumors, the growth rate of a mouse triple-negative breast cancer cell line, 4T1, was measured in 8-week-old female BALB/c mice (eight mice in each group). After two weeks of injection of DMSO or Sephin1, each BALB/c mouse was subcutaneously inoculated with $10^{6}$ 4T1 cells. Tumor volume was then measured every 2-3 days.

#### Isolation of mouse PBMCs

Peripheral blood samples were collected from the mouse eyes. Each sample was first mixed with 200 μl EDTA and then mixed with PBS in an equal volume. Equal volumes of Ficoll-Paque PREMIUM (Amersham/GE, 17544602) and the blood-EDTA-PBS solution were added to a 15 ml centrifuge tube and centrifuged at 400 × g for 20 min. The peripheral blood mononuclear cells (PBMCs) were collected, and the erythrocytes were removed with ACK lysing buffer (ThermoFisher, A1049201). The remaining cells were filtered with a 30 μm MACS SmarterStrainer (Miltenyi/MACS, 130-110-915), washed with PBS 1-2 times, and resuspended in PBS to a proper volume. The cells were stained with AO/PI (Nexcelom Bioscience, CS2-0106-5mL) and quantified using a Cellometer K2 (Nexcelom Bioscience).

#### Tumor tissue processing

Tumor tissues were collected and cut into small pieces (approximately 1-2 mm). Then, we digested the tumor tissues with a mouse tumor dissociation kit (Miltenyi/MACS, 130-096-730) following the standard procedure. After that, the cell suspension was filtered with a 30 μm MACS SmarterStrainer. Then, we performed either FACS analysis or FACS sorting (sorting for CD45^+^ and live cells).

#### FACS sorting and analysis

The FACS sorting procedure was performed before single-cell library construction with immune cells in tumor samples. The tumor cell suspensions were first incubated with a mouse CD45-specific antibody (BioLegend, 157607) for 30 minutes and then incubated with propidium iodide solution (Nexcelom Bioscience, CS1-0109-5mL). Cells were sorted on a BD SORP FACSAria.

FACS analysis was performed on tumor tissues. After single-cell suspensions were obtained, each sample was first stimulated with Cell Activation Cocktail with Brefeldin A (BioLegend, 423303) at a cell density of approximately $5\times 10^{6}$ cells/mL, and the volume ratio of the cocktail and the cell suspension was 1:500. After 4 hours of stimulation at 37 °C, the cells were centrifuged at 400 × g for 7 min, the supernatant was discarded, and then FACS antibodies were incubated with the samples. The cells were first incubated with mouse surface antibodies, including CD45 (Thermo Fisher, 12-0451-83; BioLegend, 103105; BioLegend, 157607), CD3E (BD Pharmingen, 553064), CD4 (BioLegend, 100548), CD8A (Thermo Fisher, 25-0081-81), NK1.1 (Thermo Fisher, 48-5941-80), FOXP3 (BioLegend, 126419), PD-1 (Thermo Fisher, 17-9985-82), CD11b (BioLegend, 101215), F4/80 (BioLegend, 123125), TCRβ (BioLegend, 109205) and reagents from a LIVE/DEAD Viability Kit (ThermoFisher, L34994/L34963) for 30 min. After that, the cells were centrifuged at 1500 rpm for 5 min and washed with PBS once. A Cytofix/Cytoperm Kit (554714, BD Pharmingen) was then used for cell fixation and permeabilization. Then, the cells were washed and incubated with a mouse IFNG-specific antibody (BioLegend, 505806) for 30 min. After that, the cells were washed and resuspended in BD Perm/Wash buffer from the Cytofix/Cytoperm Kit. The prepared cell suspensions were analyzed on a CytoFLEX LX from Beckman Coulter.

#### Library construction and sequencing for single-cell RNA-seq analysis

Twelve samples were used for single-cell library construction. First, after two weeks of DMSO or Sephin1 injections, we randomly selected two mice in each group and collected all four PBMC samples. Second, two weeks after B16F1 cell injection, we randomly selected two additional mice from each group and collected four PBMC samples and four tumor immune cell samples (CD45^+^ cells isolated from tumor tissues by FACS).

The cell suspension samples we obtained with this procedure were used for single-cell library construction. We performed single-cell immune profiling following the standard procedure from 10X Genomics. The library construction kit we used included the Chromium Next GEM Single Cell 5’ Library & Gel Bead Kit v1.1 (16 rxns, PN-1000165), Chromium Single Cell 5’ Library Construction Kit (16 rxns, PN-1000020), Chromium Single Cell V(D)J Enrichment Kit (Mouse T cell, 96 rxns, PN-1000071), Chromium Next GEM Chip G Single Cell Kit (48 rxns, PN-1000120) and Single Index Kit T Set A (96 rxns, PN-1000213). After single-cell library construction, all samples were sequenced on an Illumina NovaSeq PE150 platform. We obtained 12 5’ gene expression libraries and 12 matched TCR-enriched libraries. All the gene expression libraries were sequenced with a data size of 80 G. The data size of each TCR-enriched library was 10 G.

#### Single-cell data processing and integration with TCR enrichment data

After obtaining all 12 expression sequencing datasets and 12 TCR enrichment sequencing datasets, we first analyzed the data using CellRanger (version 6.0.0) software from 10X Genomics. The single-cell expression data were then imported into R and integrated with Seurat (version 3.2.3). To minimize information loss and filter out low-quality and duplicated cells at the same time, genes expressed in at least 2 cells were kept, and cells with more than 100 but less than 4000 genes were kept. In addition, violin plot was made by the percentage of mitochondrial genes, and cells that were discrete in the violin plot were filtered out. The filtered cells were then integrated, normalized, scaled and clustered with Seurat. Cell type annotation was then performed using classic immune cell markers.

The filtered TCR contig matrix was analyzed and integrated using scRepertoire (version 1.2.1)^83^ and then integrated with the gene expression data. TCRs were separated and annotated by their distribution in one sample. If the percentage of the clone number of one clonotype in all clones of the sample was between 0.1 and 1, the clonotype was classified as “hyperexpanded”; if the percentage was between 0.01 and 0.1, the classification was “large”. “medium” was used to denote a percentage between 0.001 and 0.01, and “small” indicated a percentage between $10^{-4}$ and 0.001. Separated TCRs were then integtated with the expression data. The integration process was scripted and performed on Python (version 2.7.5) and R (version 3.6.3) platforms. Cells were matched between the TCR enrichment data and expression data according to their specific barcode sequence. Plots of different TCR types were made with ggplot2 (version 3.3.5).

#### Regulon analysis by SCENIC

SCENIC (version 1.1.2-01)^51^ analysis was also performed to analyze the activity of important transcription factors and their related genes. First, 5000 cells from all 12 samples were randomly selected to identify the coexpression network with higher activities using GENIE3 (version 1.8.0). After that, SCENIC analysis was performed on all cells, and specific regulons were filtered from the coexpression network. Then, we calculated the activities of different regulons in different sample types and cell types. Besides, the regulon specificity score (RSS) of each sample was also calculated^84^ in order to identify the sample-specific regulons of each sample type.

#### Analysis of differentially expressed gene patterns between clusters

Differentially expressed genes in different clusters or samples were identified with the FindMarkers package of Seurat. The differentially expressed genes were then used to perform enrichment analyses, including Gene Set Variation Analysis (GSVA) and Gene Set Enrichment Analysis (GSEA), which were completed with the R packages GSVA (version 1.30.0) and fgsea (version 1.8.0). In addition, the AddModuleScore package of Seurat was also used to analyze the expression activities of genes involved in important pathways related to antitumor immunity. All the processes were completed on Python (version 2.7.5) and R (version 3.6.3) platforms. Plots were made with the ggplot2 (version 3.3.5), ggpubr (version 0.4.0), pheatmap (version 1.0.12) and ComplexHeatmap (version 2.8.0) packages in R.

#### Cell-cell communication analysis

Cell-cell communication analysis were completed mainly with the R package CellChat (version 1.0.0).^56^ Five cell types that played important roles in the antitumor immunity were chosen for communication analysis. The communication numbers and strengths between the normal and Sephin1 groups in different tissues were calculated and compared.

#### *In vitro* analysis of the effect of Sephin1 on mouse CD8^+^ T cells

A round-bottom 96-well plate was first prepared by incubation with 100 μl PBS supplemented with 1 μg/mL anti-mouse CD3ε (BioXCell, BE0001-1-5MG) and anti-mouse CD28 (BioXCell, BE0015-1-5MG) the day before CD8^+^ T-cell isolation. The supernatant was discarded before use.

CD8^+^ T cells were isolated from the spleen tissue of adult male C57BL/6 mice with a MojoSort Mouse CD8 T Cell Isolation Kit (BioLegend, 480035) according to the standard protocol. The isolated CD8^+^ T cells were first incubated with reagents from a CFSE Cell Division Tracker Kit (BioLegend, 423801) according to the standard protocol and then resuspended in RPMI 1640 medium (Gibco, 11875093) supplemented with 10% FBS (Biological Industries, 04-001-1A) and 1% Pen Strep (Gibco, 15140122). Then, 20 ng/mL mouse IL2 (Novoprotein, CK24) and IL7 (Novoprotein, CC73) were added, and the concentration of cells was 10^6^/mL.

The isolated CD8^+^ T cells were then incubated in the precoated 96-well plates for 72 hours. After that, the cells were collected and stained with a LIVE/DEAD Fixable Violet Dead Cell Stain Kit (Invitrogen, L34963), PerCP/Cyanine 5.5-conjugated anti-mouse CD8a antibody (BioLegend, 100733) and PE-conjugated anti-mouse IFN-γ antibody (BioLegend, 163503) as described above. The prepared cell suspensions were analyzed on a CytoFLEX LX from Beckman Coulter.

#### Immunofluorescence analysis

Immunofluorescence analysis was performed with mouse antibodies of F4/80 (Servicebio, GB11027), CD44 (Servicebio, GB112054), FN1 (Servicebio, GB112093) and SPP1 (Servicebio, GB11500). The tumor tissue was firstly fixed with paraformaldehyde, and embedded in paraffin, and then used for immunofluorescence staining.

### Quantification and statistical analysis

All statistical analyses were conducted using GraphPad Prism 7 (La Jolla, CA, USA) or R (version 3.6.3). Statistic differences between groups were calculated with different methods depended on the data characteristics, including unpaired two-tailed Student’s t-test, Chi square test, or Wilcoxon test (annotated in the figure legends). Statistical parameters are either shown with exact p values or as: ∗: p < 0.05, ∗∗: p < 0.01, ∗∗∗: p < 0.001, ∗∗∗∗: p < 0.0001.

## Data Availability

• Single-cell RNA-seq data have been deposited at GEO and are publicly available as of the date of publication. The accession number is GEO: GSE220656. The original codes are deposited in github with this link: GitHub: https://github.com/LisaWang2022/B16F1-mouse-single-cell-analysis • Any additional information required to reanalyze the data reported in this paper is available from the lead contact upon request.

## Supplemental information

Document S1. Figures S1–S11

Table S1. Single-cell metrics (Information of single-cell metrics before/after filtering by Seurat, including cell number, median genes/UMI counts per cell and fraction reads in cells, related to Figure 1

Table S2. AddModuleScore gene sets (Gene sets used for AddModuleScore function, related to Figures 3, 5, and 6

Table S3. Adjusted p values (p values before/after adjustment between groups, related to Figures 3, 5, and 6
