# Supplementary material for: Single-cell RNA sequencing reveals the suppressive effect of PPP1R15A inhibitor Sephin1 in antitumor immunity
Source: iScience. 2023 Jan 13;26(2):105954. doi: 10.1016/j.isci.2023.105954 (PMC9883195; doi:10.1016/j.isci.2023.105954)
Supplement: Document S1. Figures S1–S11 [file mmc1.pdf]

iScience, Volume *26*

Supplemental information

Single-cell RNA sequencing reveals the suppressive effect of PPP1R15A inhibitor Sephin1 in antitumor immunity

Rongjing Wang, Yuchao Zhang, Shiwei Guo, Siyu Pei, Wei Guo, Zhenchuan Wu, Hailong Wang, Minghui Wang, Yizhe Li, Yufei Zhu, Ling-Hua Meng, Jingyu Lang, Gang Jin, Yichuan Xiao, Landian Hu, and Xiangyin Kong

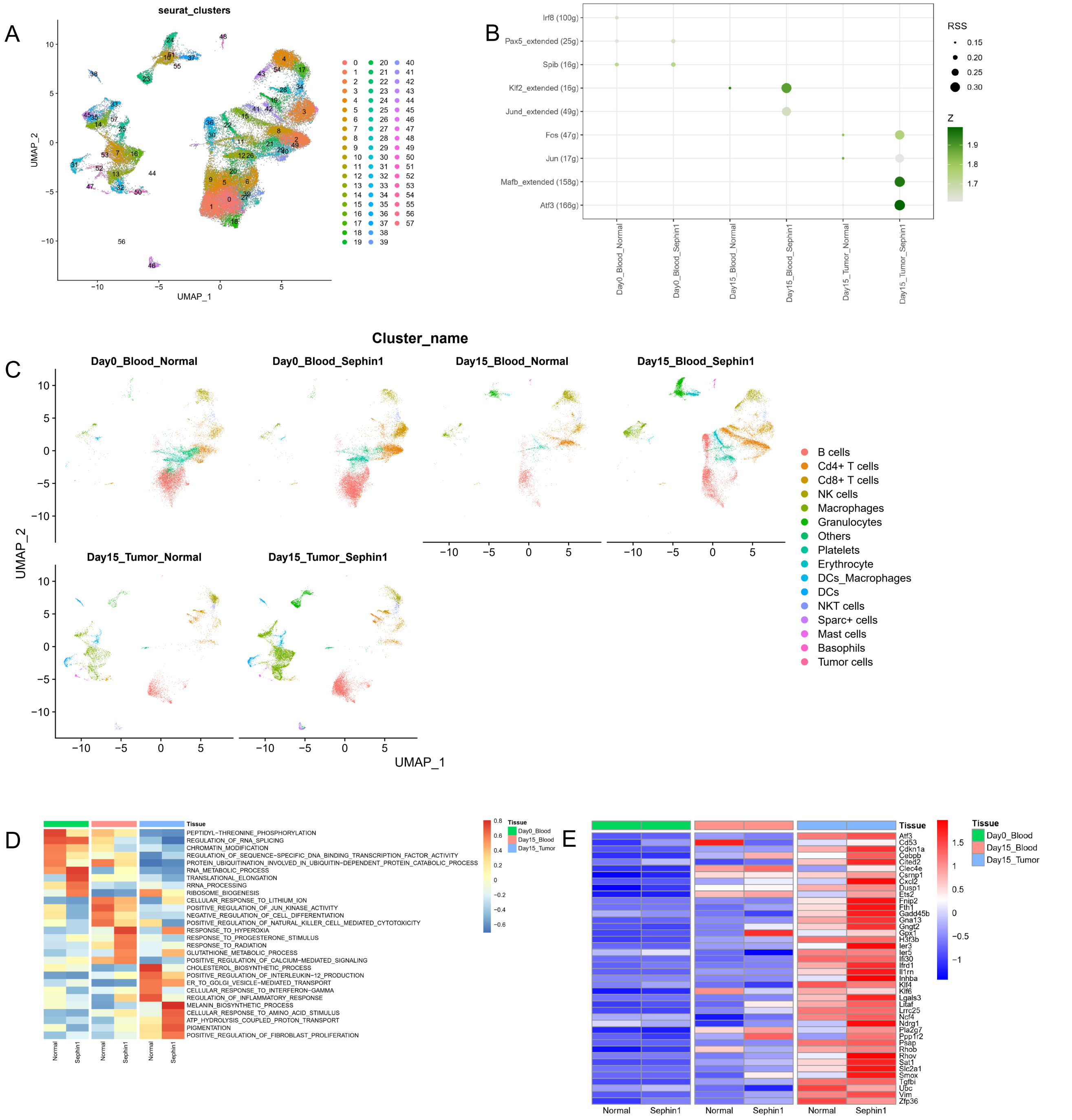

Figure S1. Distribution and expression patterns of different cell types, related to Figure 1.

(A) Original Seurat clusters of all samples. (B) Cell-type specific regulators calculated based on the Regulon Specificity Score (RSS). (C) UMAP plot of all samples split by sample type. (D) GSVA of differentially expressed genes between samples. (E) Enriched genes from Atf3 regulon in the Sephin1 group either in the blood of day 0 and day 15, or in the tumor tissue of day 15.

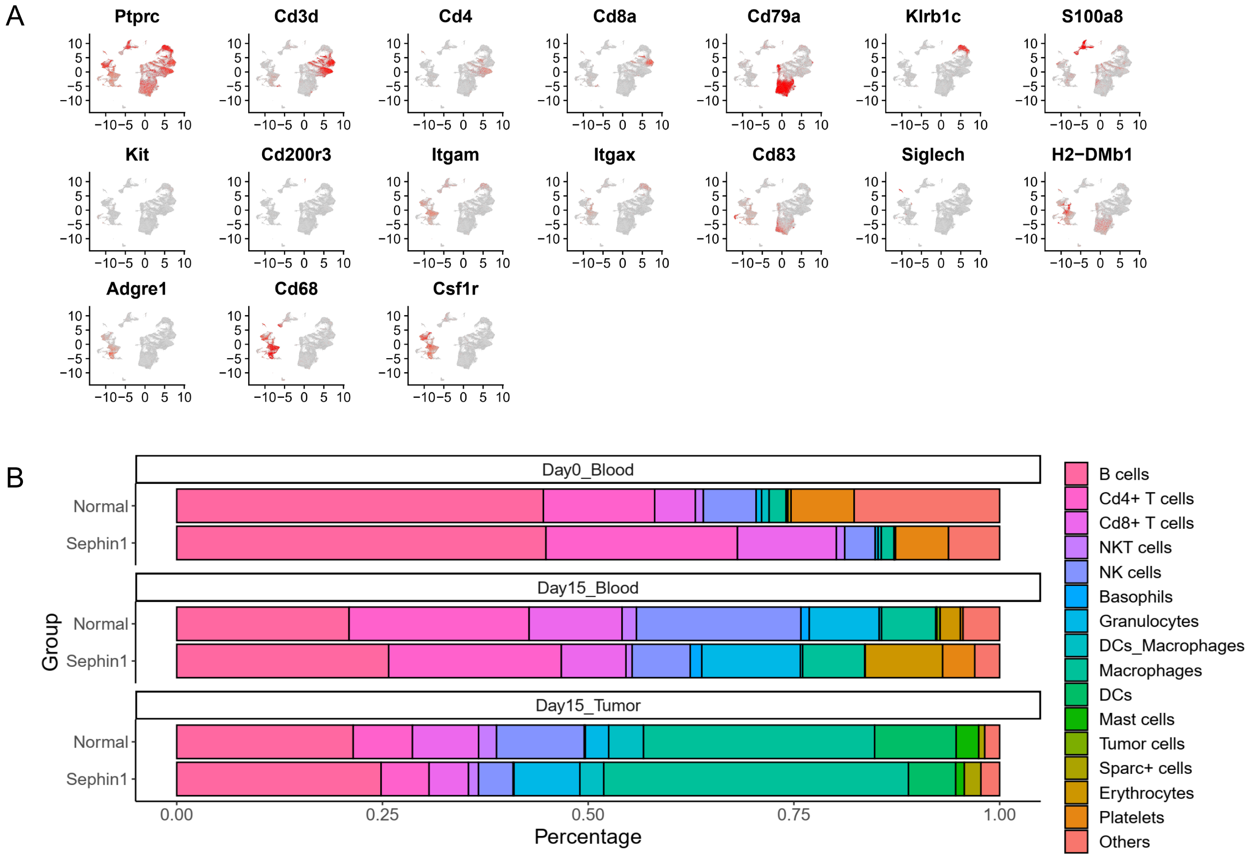

Figure S2. Distribution patterns and cell markers of different cell types, related to Figure 1.

(A) Percentages of different cell types in different sample types. (B) Gene markers used for cell type annotation. Ptprc—immune cells. Cd3d, Cd4, Cd8a—T cells. Cd79a—B cells. Klrb1c—NK cells. S100a8—granulocytes. Kit—mast cells. Cd200r3—basophils. Cd68, Csf1r, Adgre1—macrophages. H2-DMb1, Cd83, Siglech—DCs.

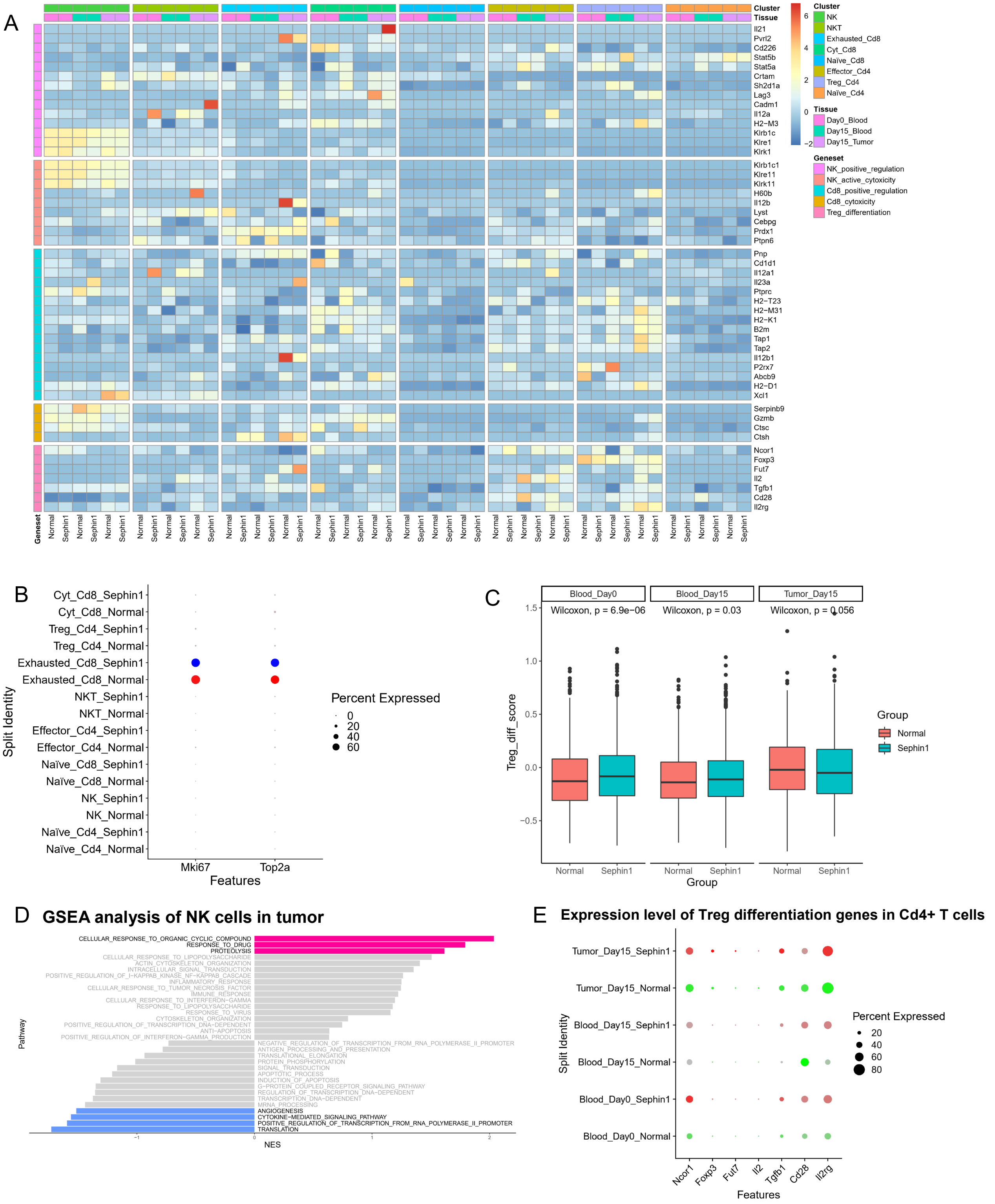

Figure S3. Expression patterns of lymphocytes, related to Figure 3.

(A) Heatmap of gene expression generated using AddModuleScore. (B) Expression patterns of two cell cycle-related genes in lymphocyte subtypes. (C) Expression score of regulatory differentiation-related genes in the Cd4+

T cells calculated by AddModuleScore. The p values was calculated by Wilcoxon test and adjusted by Bonferroni-Holm method. (D) GSEA of the most differentially expressed genes between the normal and Sephin1 groups in NK cells. Genes in pathways related to cytokine signaling and antigen processing were downregulated. Genes related to the cellular response to stress were upregulated, which showed activation of the ISR process. (E) Expression level of regulatory differentiation-related genes mentioned in (C).

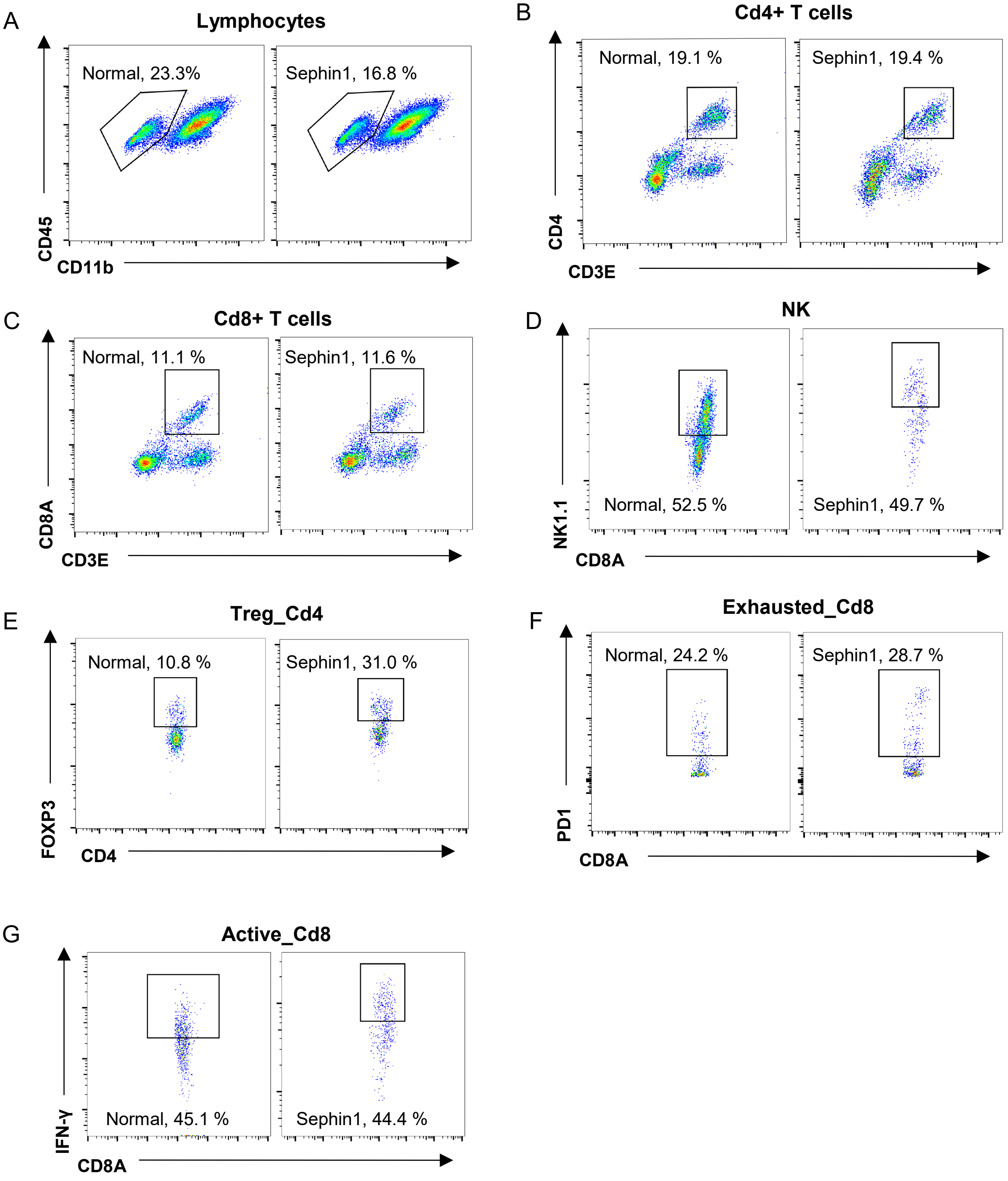

Figure S4. FACS results of antitumor-related lymphocytes in the mouse tumor tissue, related to Figure 2. Including all lymphocytes (A), Cd4+ and Cd8+ T cells (B & C), NK cells (D), Cd4+ regular T cells (E), exhausted Cd8+ T cells (F) and active Cd8+ T cells (G).

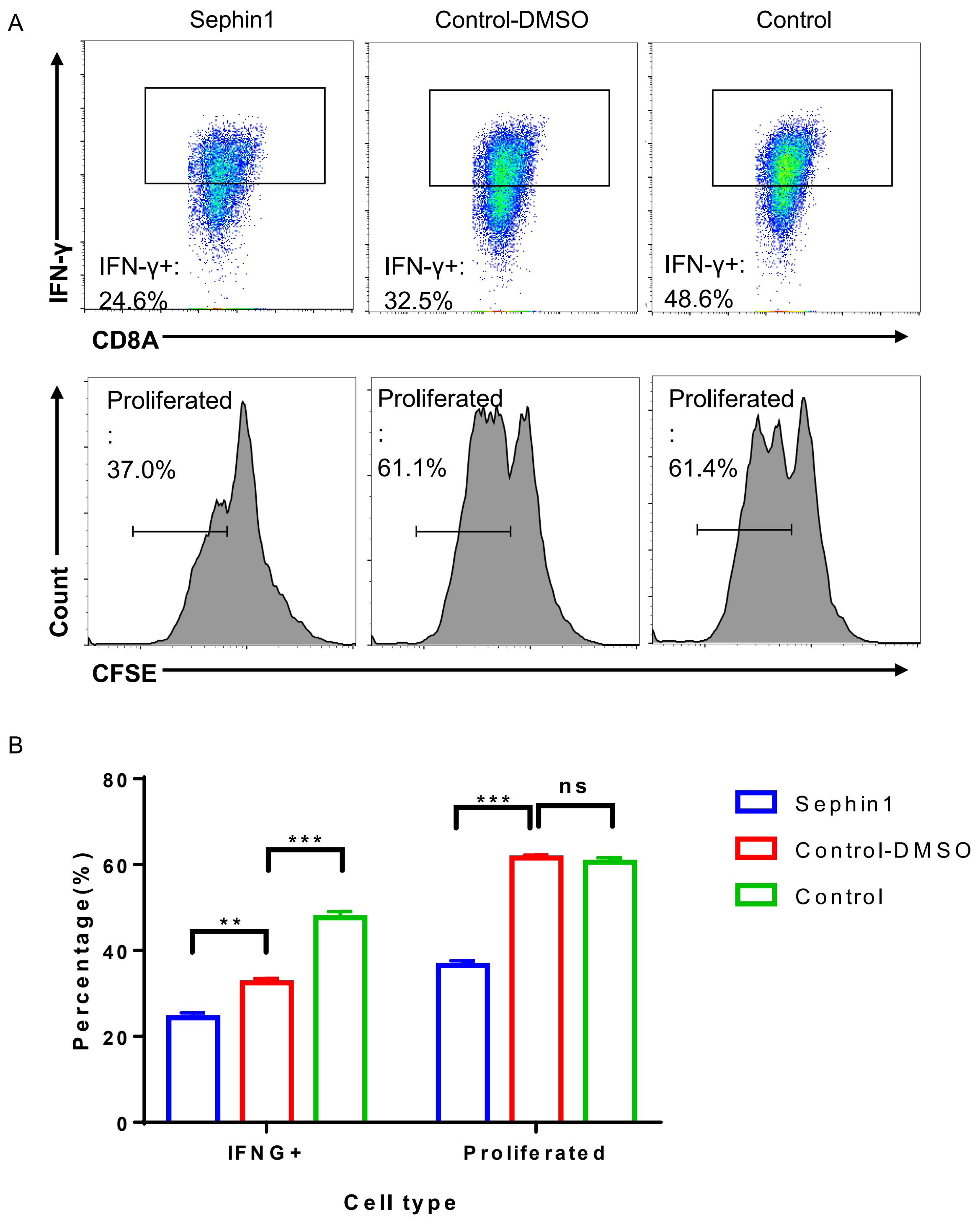

Figure S5. FACS results for the activation and proliferation of Cd8+ T cells, related to Figure 3.

(A) FACS results for IFNG and CFSE. Top: percentage of IFNG+ cells in Cd8+ T cells. Bottom: percentage of proliferated cells among Cd8+ T cells. Sephin1: Cd8+ T cells supplemented with 20 μg/mL Sephin1 and 0.2% DMSO. Control-DMSO: Cd8+ T cells treated with 0.2% DMSO. Control: Cd8+ T cells without Sephin1 or DMSO treatment. (B) Statistical analysis of the results for the Sephin1, Control-DMSO and Control groups. Cd8+ T cells

treated with Sephin1 had significantly lower expression of IFNG and a lower proliferative ability ($n = 3$). Multiple t test was used without adjustments. Bars: mean; error bars: SEM; **: $p < 0.01$; ***: $p<0.001$.

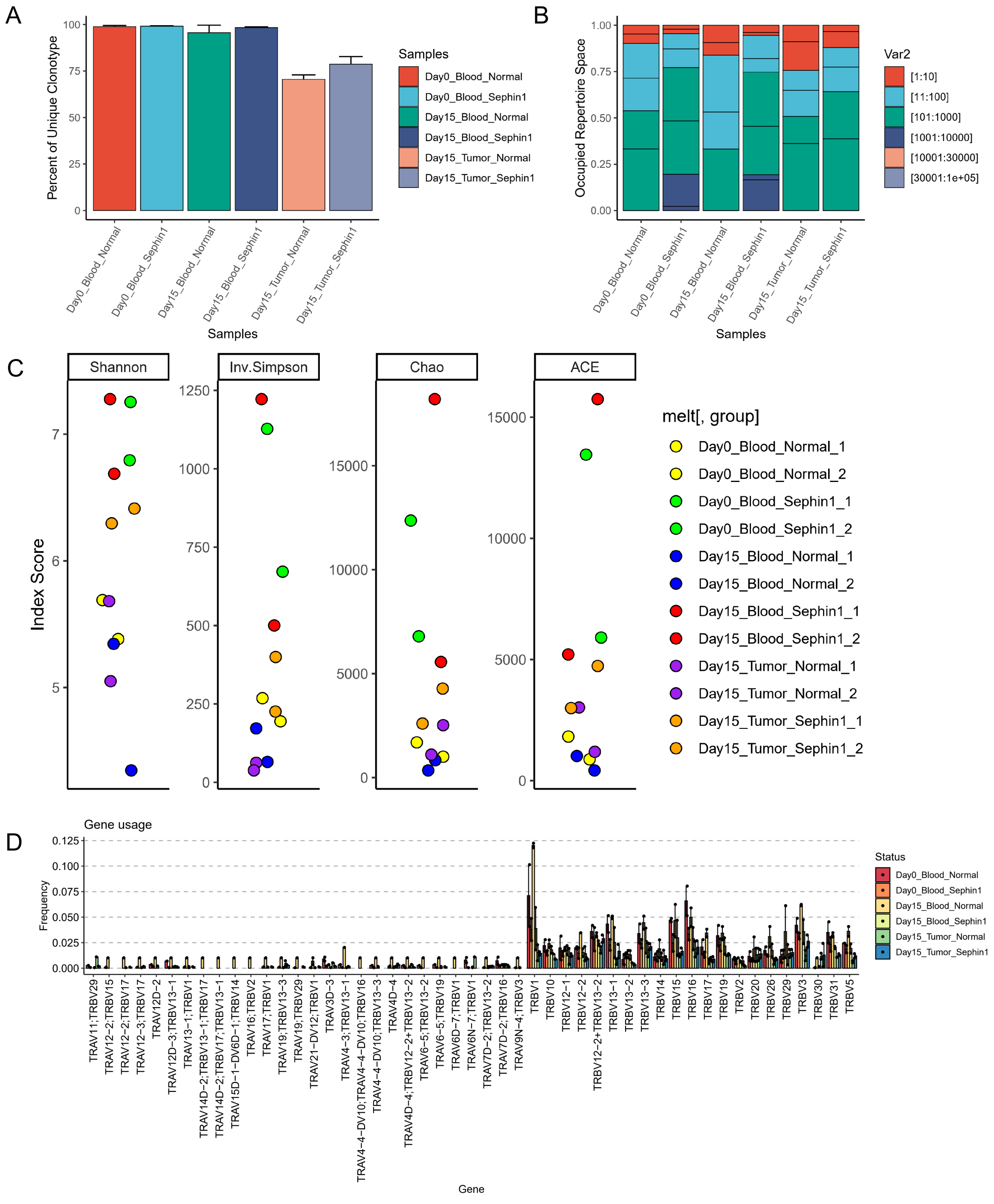

Figure S6. Expression and distribution patterns of TCR regions, related to Figure 4.

(A) Distribution of unique clonotypes. The percentage of nonunique clonotypes in the tumor microenvironment was lower than that in the blood, and the tumors in the Sephin1 group had more unique TCR clonotypes than those in the normal group. (B) Distribution of the clonal proportion in different sample types. Clonotypes in separate samples were ranked by clone number and placed into different bins. Tissues in the Sephin1 group were more enriched in low-ranking clonotypes. (C) Diversity analysis of TCR clonotypes in different sample types. The Shannon index,

inverse Simpson index, Chao1 estimator and abundance-based coverage estimator (ACE) were used for analysis. Samples in the Sephin1 group had higher TCR richness, and PBMCs had higher TCR richness than immune cells in tumors. (D) Distribution of different TCR clonotypes between samples and groups. Different samples had various TCR clonotype distribution patterns.

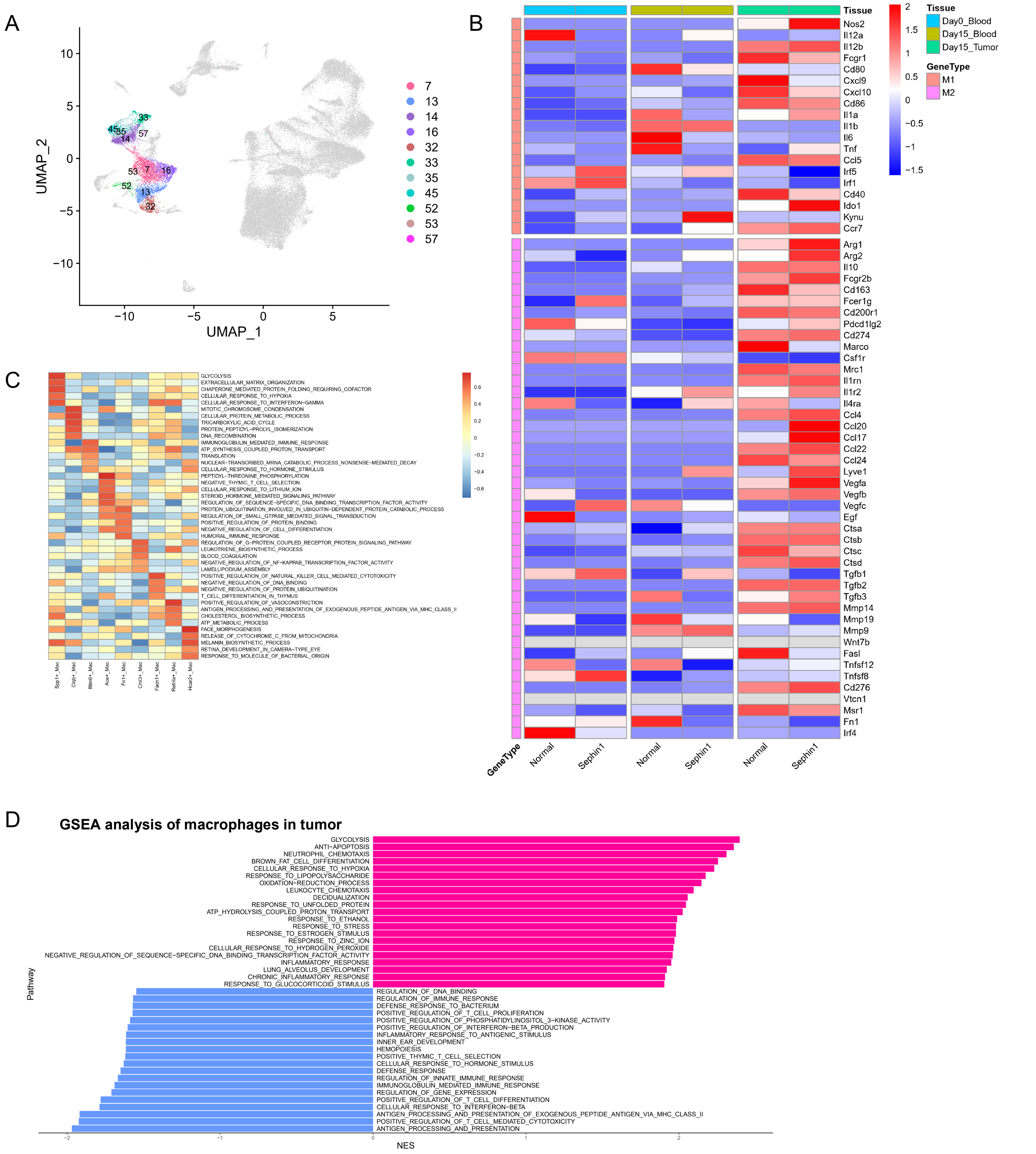

Figure S7. Expression patterns of macrophage subtypes, related to Figure 5.

(A) Seurat cluster distribution of macrophages. (B) Expression patterns of M1- and M2-related genes in different sample types. Macrophages among immune cells in tumors in the Sephin1 group were more likely to express M2-related genes. (C) GSVA of differentially expressed genes in macrophage subtypes. (D) GSEA of macrophages among immune cells in tumors between the normal and Sephin1 groups. Genes in pathways related to T-cell

activation were downregulated in the Sephin1 group.

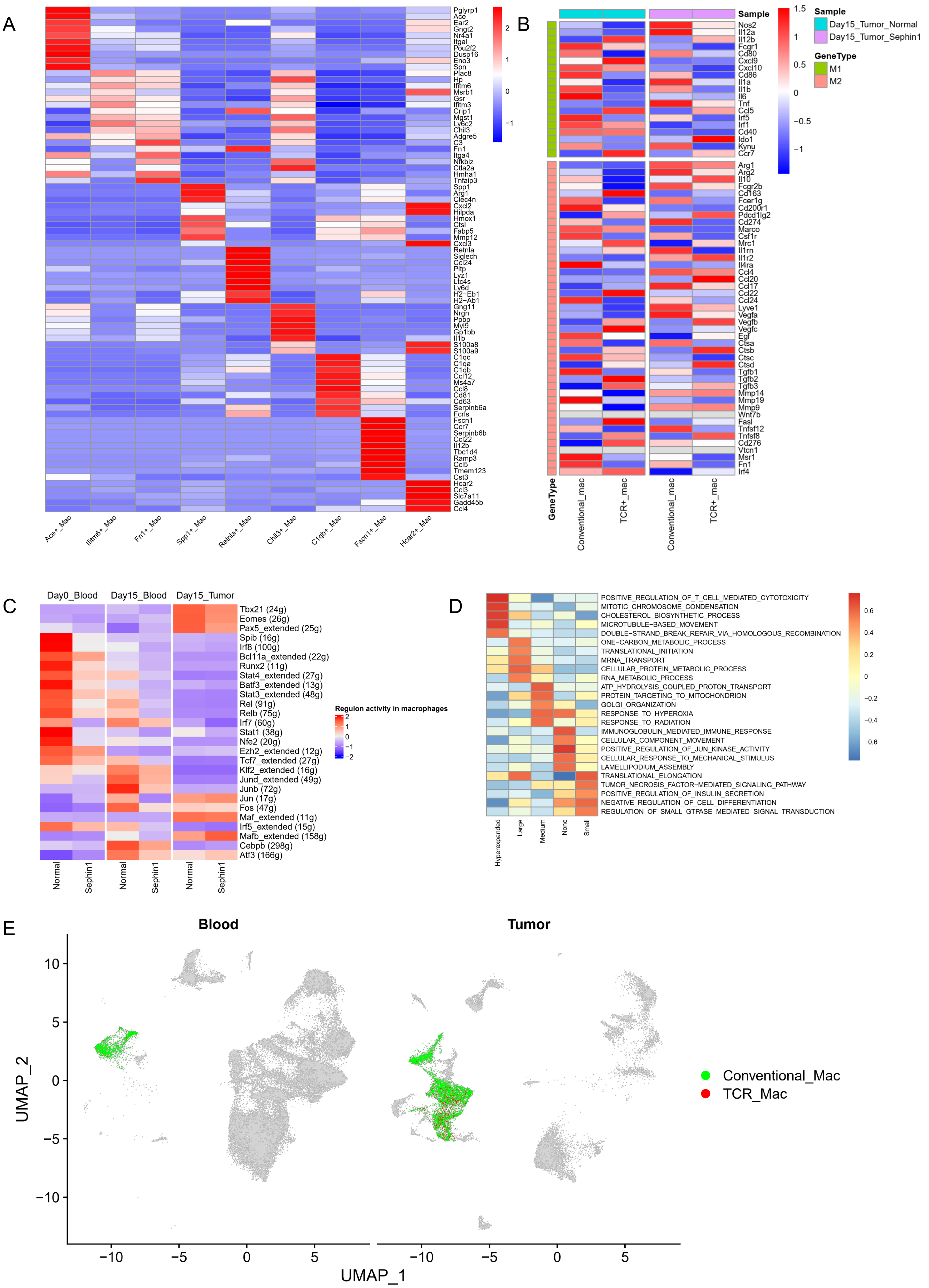
A
Pglyrp1
Ace
Ear2
Gngt2
Nr4a1
Itgal
Pou2f2
Dusp16
Eno3
Spn
Plac8
Hp
Ifitm6
Msrb1
Gsr
Ifitm3
Crip1
Mgst1
Ly6c2
Chil3
Adgre5
C3
Fn1
Itga4
Nfkbiz
Ctla2a
Hmha1
Tnfaip3
Spp1
Arg1
Clec4n
Cxcl2
Hilpda
Hmox1
Ctsl
Fabp5
Mmp12
Cxcl3
Retnla
Siglech
Ccl24
Pltp
Lyz1
Ltc4s
Ly6d
H2−Eb1
H2−Ab1
Gng11
Nrgn
Ppbp
Myl9
Gp1bb
Il1b
S100a8
S100a9
C1qc
C1qa
C1qb
Ccl12
Ms4a7
Ccl8
Cd81
Cd63
Serpinb6a
Fcrls
Fscn1
Ccr7
Serpinb6b
Ccl22
Il12b
Tbc1d4
Ramp3
Ccl5
Tmem123
Cst3
Hcar2
Ccl3
Slc7a11
Gadd45b
Ccl4
2
1
0
−1
Ace+_Mac
Ifitm6+_Mac
Fn1+_Mac
Spp1+_Mac
Retnla+_Mac
Chil3+_Mac
C1qb+_Mac
Fscn1+_Mac
Hcar2+_Mac
B
Sample
Nos2
Il12a
Il12b
Fcgr1
Cd80
Cxcl9
Cxcl10
Cd86
Il1a
Il1b
Il6
Tnf
Ccl5
Irf5
Irf1
Cd40
Ido1
Kynu
Ccr7
Arg1
Arg2
Il10
Fcgr2b
Cd163
Fcer1g
Cd200r1
Pdcd1lg2
Cd274
Marco
Csf1r
Mrc1
Il1rn
Il1r2
Il4ra
Ccl4
Ccl20
Ccl17
Ccl22
Ccl24
Lyve1
Vegfa
Vegfb
Vegfc
Egf
Ctsa
Ctsb
Ctsc
Ctsd
Tgfb1
Tgfb2
Tgfb3
Mmp14
Mmp19
Mmp9
Wnt7b
Fasl
Tnfsf12
Tnfsf8
Cd276
Vtcn1
Msr1
Fn1
Irf4
GeneType
Conventional_mac
TCR+_mac
Conventional_mac
TCR+_mac
1.5
1
0.5
0
−0.5
−1
Sample
Day15_Tumor_Normal
Day15_Tumor_Sephin1
GeneType
M1
M2
C
Day0_Blood
Day15_Blood
Day15_Tumor
Tbx21 (24g)
Eomes (26g)
Pax5_extended (25g)
Spib (16g)
Irf8 (100g)
Bcl11a_extended (22g)
Runx2 (11g)
Stat4_extended (27g)
Batf3_extended (13g)
Stat3_extended (48g)
Rel (91g)
Relb (75g)
Irf7 (60g)
Stat1 (38g)
Nfe2 (20g)
Ezh2_extended (12g)
Tcf7_extended (27g)
Klf2_extended (16g)
Jund_extended (49g)
Junb (72g)
Jun (17g)
Fos (47g)
Maf_extended (11g)
Irf5_extended (15g)
Mafb_extended (158g)
Cebpb (298g)
Atf3 (166g)
Regulon activity in macrophages
2
1
0
−1
−2
Normal
Sephin1
Normal
Sephin1
Normal
Sephin1
D
POSITIVE_REGULATION_OF_T_CELL_MEDIATED_CYTOTOXICITY
MITOTIC_CHROMOSOME_CONDENSATION
CHOLESTEROL_BIOSYNTHETIC_PROCESS
MICROTUBULE−BASED_MOVEMENT
DOUBLE−STRAND_BREAK_REPAIR_VIA_HOMOLOGOUS_RECOMBINATION
ONE−CARBON_METABOLIC_PROCESS
TRANSLATIONAL_INITIATION
MRNA_TRANSPORT
CELLULAR_PROTEIN_METABOLIC_PROCESS
RNA_METABOLIC_PROCESS
ATP_HYDROLYSIS_COUPLED_PROTON_TRANSPORT
PROTEIN_TARGETING_TO_MITOCHONDRION
GOLGI_ORGANIZATION
RESPONSE_TO_HYPEROXIA
RESPONSE_TO_RADIATION
IMMUNOGLOBULIN_MEDIATED_IMMUNE_RESPONSE
CELLULAR_COMPONENT_MOVEMENT
POSITIVE_REGULATION_OF_JUN_KINASE_ACTIVITY
CELLULAR_RESPONSE_TO_MECHANICAL_STIMULUS
LAMELLIPODIUM_ASSEMBLY
TRANSLATIONAL_ELONGATION
TUMOR_NECROSIS_FACTOR−MEDIATED_SIGNALING_PATHWAY
POSITIVE_REGULATION_OF_INSULIN_SECRETION
NEGATIVE_REGULATION_OF_CELL_DIFFERENTIATION
REGULATION_OF_SMALL_GTPASE_MEDIATED_SIGNAL_TRANSDUCTION
0.6
0.4
0.2
0
−0.2
−0.4
−0.6
Hyperexpanded
Large
Medium
None
Small
E
Blood
Tumor
UMAP_2
10
5
0
−5
−10
−10
−5
0
5
−10
−5
0
5
UMAP_1
Conventional_Mac
TCR_Mac

Figure S8. Expression patterns of macrophage subtypes and TCR+ macrophages, related to Figure 6.
(A) Most highly expressed genes in macrophage subtypes. The top 10 genes in each subgroup are shown. (B) Expression level of M1 and M2 related genes in TCR+ macrophages and conventional macrophages in the tumor tissue. (C) SCENIC analysis of macrophages among sample types. The Atf3 regulon was upregulated in the Sephin1 group in Day0_Blood and Day15_Tumor samples. (D) GSVA of different TCR types in TCR+ macrophages. The hyperexpanded cluster was more enriched in pathways related to T cell positive regulation, mitotic chromosome condensation, cholesterol biosynthetic process, microtubule-based movement and double-strand break repair via homologous recombination. (E) Distribution of TCR+ and conventional macrophages in the normal group and Sephin1 group.

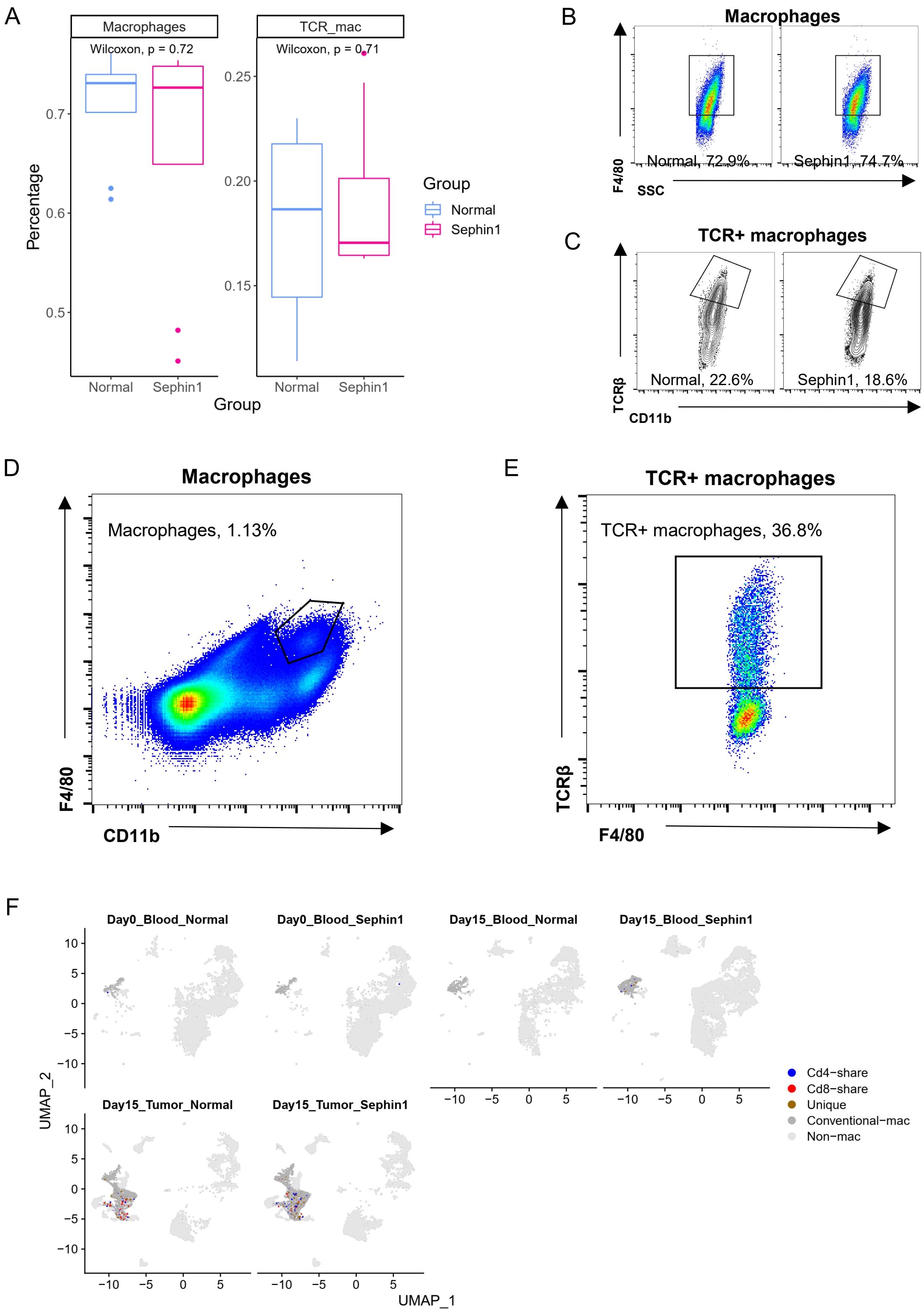

Figure S9. Distribution of TCR+ macrophages in tumor samples and spleens, related to Figure 6.

(A) Percentage of macrophages in all myeloid cells and TCR+ macrophages in all macrophages in the tumor

microenvironment determined by FACS (n = 8). Wilcoxon test was used in each cell type without adjustment and p values was marked in the graph. (B-C) FACS results of macrophages and TCR+ macrophages. (D-E) FACS results of TCR+ macrophages in the spleen tissue of normal mice. (F) Distribution of macrophages having shared TCRs with T cells in different samples.

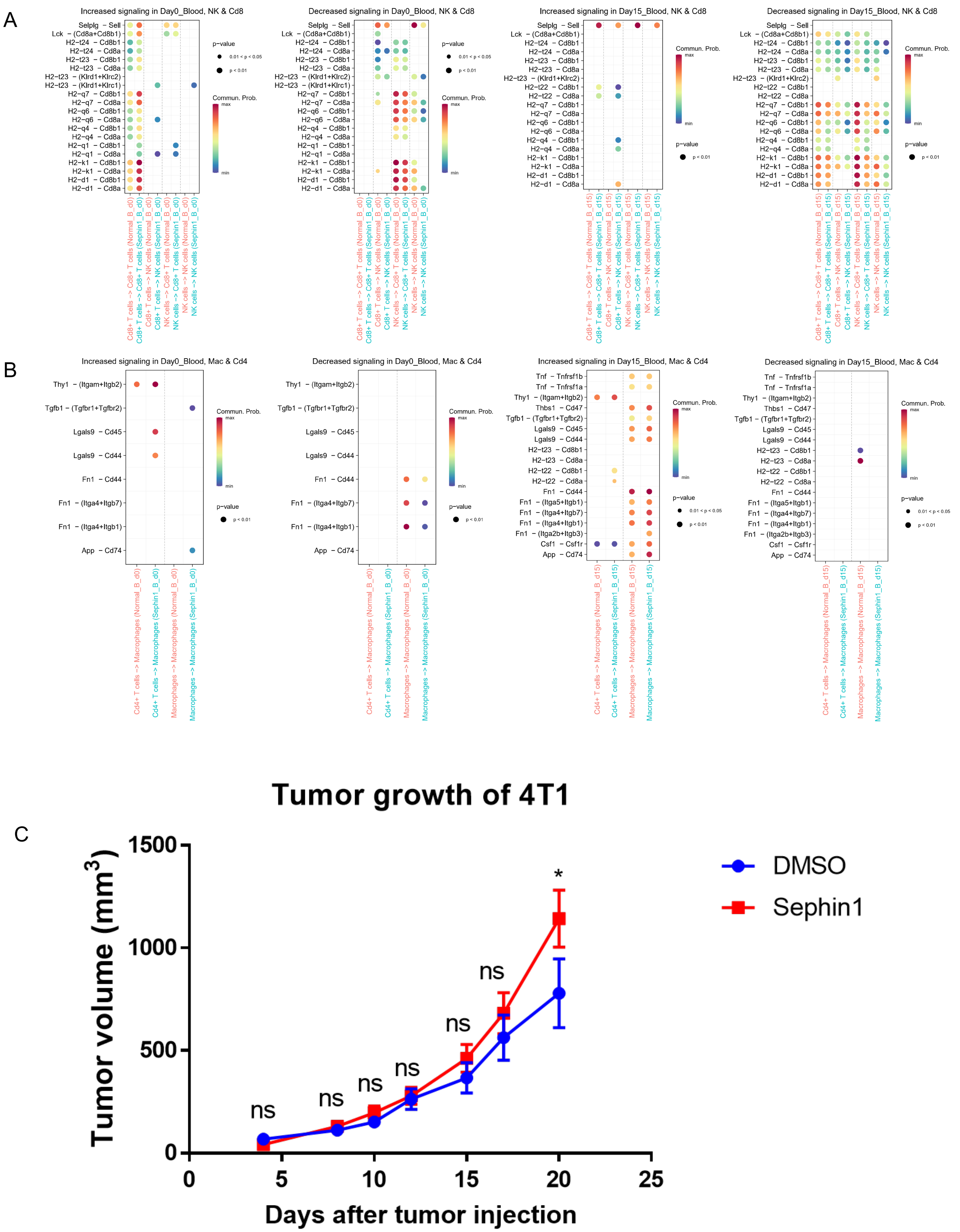
A
Increased signaling in Day0_Blood, NK & Cd8
Decreased signaling in Day0_Blood, NK & Cd8
Increased signaling in Day15_Blood, NK & Cd8
Decreased signaling in Day15_Blood, NK & Cd8
Commun. Prob.
p-value
B
Increased signaling in Day0_Blood, Mac & Cd4
Decreased signaling in Day0_Blood, Mac & Cd4
Increased signaling in Day15_Blood, Mac & Cd4
Decreased signaling in Day15_Blood, Mac & Cd4
C
Tumor growth of 4T1
Tumor volume (mm3)
Days after tumor injection
DMSO
Sephin1
ns
*

Figure S10. Ligand-receptor results in blood and tumor growth results of 4T1, related to Figure 7.
(A) Ligand-receptor pairs of mostly downregulated pathways in the Sephin1 group in the blood of day 0 and day 15 between Cd8+ T cells and NK cells. (B) Ligand-receptor pairs of mostly upregulated pathways in the Sephin1 group in the blood of day 0 and day 15 between Cd4+ T cells and macrophages. (C) Tumor growth of 4T1 cells in female BALB/c mice ($n = 8$). Multiple t test was used without adjustments, and each row was analyzed individually. Bars: mean; error bars: SEM; *: $p < 0.05$. The tumor growth rate in the Sephin1 group was higher than that in the normal group. However, the result was not as significant as that for B16F1 cells in male C57BL/6 mice.

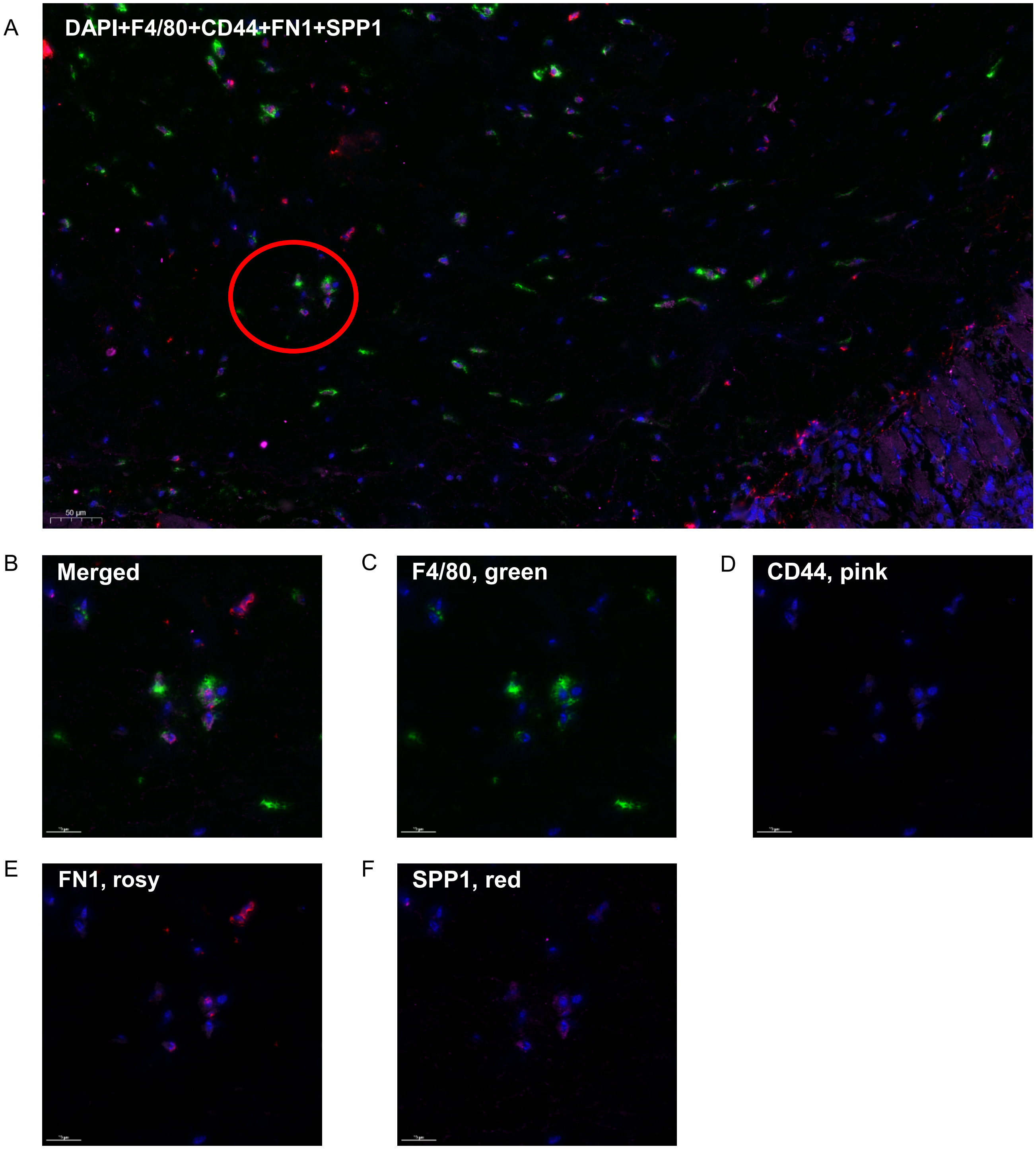

Figure S11. Immunofluorescence results of ligand-receptor expression in macrophages of day 15 tumor tissue, related to Figure 7.

(A) Merged results of DAPI, F4/80, CD44, FN1 and SPP1, 50μm. (B-F) Merged and separated results of each molecule. 20μm.
